# Polyphenolic grape stalk and coffee extracts attenuate spinal cord injury-induced neuropathic pain development in ICR-CD1 female mice

**DOI:** 10.1038/s41598-022-19109-4

**Published:** 2022-09-02

**Authors:** Anna Bagó-Mas, Andrea Korimová, Meritxell Deulofeu, Enrique Verdú, Núria Fiol, Viktorie Svobodová, Petr Dubový, Pere Boadas-Vaello

**Affiliations:** 1 Research Group of Clinical Anatomy, Embryology and Neuroscience (NEOMA), Department of Medical Sciences, University of Girona, Girona, Spain; 2 Department of Anatomy, Division of Neuroanatomy, Faculty of Medicine, Masaryk University, Brno, Czechia; 3 Department of Chemical Engineering, Agriculture and Food Technology, Polytechnic School, University of Girona, Girona, Spain

**Keywords:** Diseases of the nervous system, Sensory processing, Preclinical research, Spinal cord diseases

## Abstract

More than half of spinal cord injury (SCI) patients develop central neuropathic pain (CNP), which is largely refractory to current treatments. Considering the preclinical evidence showing that polyphenolic compounds may exert antinociceptive effects, the present work aimed to study preventive effects on SCI-induced CNP development by repeated administration of two vegetal polyphenolic extracts: grape stalk extract (GSE) and coffee extract (CE). Thermal hyperalgesia and mechanical allodynia were evaluated at 7, 14 and 21 days postinjury. Then, gliosis, ERK phosphorylation and the expression of CCL2 and CX3CL1 chemokines and their receptors, CCR2 and CX3CR1, were analyzed in the spinal cord. Gliosis and CX3CL1/CX3CR1 expression were also analyzed in the anterior cingulate cortex (ACC) and periaqueductal gray matter (PAG) since they are supraspinal structures involved in pain perception and modulation. GSE and CE treatments modulated pain behaviors accompanied by reduced gliosis in the spinal cord and both treatments modulated neuron-glia crosstalk-related biomolecules expression. Moreover, both extracts attenuated astrogliosis in the ACC and PAG as well as microgliosis in the ACC with an increased M2 subpopulation of microglial cells in the PAG. Finally, GSE and CE prevented CX3CL1/CX3CR1 upregulation in the PAG, and modulated their expression in ACC. These findings suggest that repeated administrations of either GSE or CE after SCI may be suitable pharmacologic strategies to attenuate SCI-induced CNP development by means of spinal and supraspinal neuroinflammation modulation.

## Introduction

Central neuropathic pain (CNP) following spinal cord injury (SCI) develops in more than half of patients^1^, and approximately one-third of those report the pain to be severe^2,3^. Traumatic SCI results in the disruption of efferent and afferent pathways, leading to various sensorimotor deficits at or below the level of injury^4^. CNP resulting from SCI involves a complex pathophysiology along the entire neuroaxis leading to central sensitization, including neuronal and glial changes, neuroinflammation, dysregulation of descending inhibitory pathways, upregulation of ascending facilitation and spinal hyperexcitability^5^. Among these phenomena, gliosis may play a pivotal role in CNP development. Actually, it is well known that glial activation contributes to the development of CNP, as reactive glial cells secrete proinflammatory cytokines, chemokines, prostaglandins, nitric oxide, and neuropeptides/neurotrophins (e.g., CGRP, SP, BDNF, NGF), which contribute to the sensitization of spinal nociceptive neurons, facilitating the release of neurotransmitters by dorsal horn nociceptive afferents on spinal neurons and depolarizing spinal nociceptive neurons. All these phenomena activated by chemical mediators secreted by reactive glial cells cause hyperexcitability of nociceptive neurons and pain^6^.

Several pharmacological treatments have been used to alleviate CNP, but current drugs are usually inadequate, and only one-third of patients respond to pharmacological treatments when compared with placebo^7^. Hence, given the lack of effective treatments, it is necessary to develop new pharmacological strategies not only for the relief of neuropathic pain but also for the prevention of its chronification. Current strategies aimed at modulating pathological pain include the use of polyphenols, as preclinical evidence of their antinociceptive effects can be found in the literature^8^. Among the properties that can be attributed to polyphenols and that may explain pain modulation are free radical scavenging/antioxidant, immunomodulatory, neuroprotective, anti-apoptotic and autophagy-regulating activities^8^. However, although several studies have been specifically aimed at elucidating the effects of polyphenolic treatments on the development of neuropathic pain, most of them have been conducted in preclinical models unrelated to SCI, such as peripheral neuropathic pain^9,10^, among others. Furthermore, although few studies on polyphenol treatment after SCI are available, most of them have focused on motor recovery or spinal cord regeneration^11–14^, leading to a lack of information despite promising results on their effects on modulating pathophysiological processes that may also be related to the induction of neuropathic pain. Thus, while there is a wealth of data on the beneficial effects of polyphenolic treatments, few studies have demonstrated the modulation of SCI-induced neuropathic pain by polyphenols^15–18^.

In this context, the present work aimed to study the preventive effects of the administration of two vegetal polyphenolic extracts on central neuropathic pain development in spinal cord-injured mice. The two polyphenolic extracts were obtained from grape residual material (GSE) and roasted decaffeinated coffee powder (CE) since both grapevine and coffee are known to be rich natural sources of polyphenols^19,20^. After SCI, the effects of repeated GSE and CE treatment on weight, thermal hyperalgesia, mechanical allodynia, and locomotor activity were assessed up to the end of the SCI acute phase (21 days postinjury). At the end of the experimental period, serum levels of hepatotoxicity and nephrotoxicity biomarkers were analyzed to assess the pharmacological safety of the polyphenolic treatments. Finally, to explain the potential effects exerted by these treatments on CNP behaviors, astrogliosis and microgliosis were analyzed in the spinal cord and the supraspinal structures periaqueductal gray matter (PAG), which is involved in pain modulation, and the anterior cingulate cortex (ACC), which plays an important role in both sensory and affective components of pain. Moreover, to obtain some mechanistic insights, the protein expression of ERK phosphorylation as well as the expression of CCL2 and CX3CL1 chemokines and their receptors, CCR2 and CX3CR1, were analyzed since all of them have been reported to be involved in CNP development^21–24^.

## Methods

### Preparation of polyphenolic extracts and quantification of polyphenol content in the extracts

The two polyphenolic extracts used in the present study were obtained from different natural sources. The grape stalk extract (GSE) was obtained from Grape stalks wastes of *Cabernet Sauvignon* and *Merlot* varieties, generated in the wine production process in *L’Empordà* region of Catalonia, genteelly provided by the owner of the winery *Celler Roig Parals* (Mollet de Peralada, Catalonia). The plant collection was accomplished in accordance with the national guidelines and regulations. The coffee extract (CE) was obtained from commercial decaffeinated ground roasted coffee, blend of Robusta natural (pure *Coffea canephora*) and Arabica natural (pure *Coffea arabica*) at a roast point 7 over 10 (dark medium).

To collect the GSE particles, grape stalk residual material was cut with a chopper grinder (WCG75 Pro Prep) and was then sifted with a digital electromagnetic sieve (CISA BA 200N) to achieve a particle size between 0.5 and 1 mm. For CE, processed coffee powder was used. To obtain the extracts, 3 g of either grape stalk or coffee particles were mixed with 50 mL of saline solution (0.9% Vitulia Physiological Serum, Barcelona, Spain) as a solubilizer, which was refluxed and stirred at 100 °C for 2 h. The resulting solutions were first filtered with chromatographic filters (Scharlau Nylon Syringe filter, ø 13 mm, 0.45 μm, NY13045200) and were then filtered and sterilized with 0.22 μm vacuum filter bottles (FPE-204-250, Biofil).

The total amount of polyphenols in both extracts was determined by the Folin-Ciocalteu assay. This method, also called the gallic acid equivalence method (GAE), consists of using the Folin-Ciocaiteu reagent, which is a mixture of phosphomolybdic acid (H_3_PMo_12_O_40_) and phosphotungstic acid (H_3_PW_12_O_40_) that reduces to molybdenum and tungsten oxide in the presence of phenolic and polyphenolic compounds^25^. The Folin Ciocalteu method was performed using gallic acid as a standard. The calibration curve was obtained by preparing different standard concentrations of gallic acid within the range 100–1000 mg/L. Briefly, 100 µL aliquots of diluted extracts (1:2, 1:3, 1:4), gallic acid standard solutions (100–1000 mg/L) and a blank (saline solution) were placed in different tubes. Then, 3.9 mL of Milli-Q water, 900 µL of 20% sodium carbonate and 600 µL of Folin-Ciocalteu reagent were added. The tubes were shaken and then allowed to incubate for 2 h at room temperature. After incubation, the absorbance against a blank was measured spectrophotometrically at 760 nm (Hitachi U-2000 VIS/UV spectrophotometer). The total polyphenolic content was expressed as milligrams of gallic acid equivalents (GAE) per liter of extracts.

### Characterization of phenolic compounds in the extracts

Qualitative determination of polyphenols in GSE was accomplished by high-performance liquid chromatography high resolution mass spectrometry (HPLC-HRMS) carried out by the scientific technical services of the University of Barcelona. Both GSE and CE analysis samples were prepared by filtering through a 0.45 μm syringe filter. GSE was diluted (1/5 and 1/50) with 0.1% formic acid, and the CE sample was diluted (1/10, 1/100 and 1/250) with 0.1% formic acid. Standard solutions of the identified polyphenols were used to confirm their presence and for quantification in both extracts.

HPLC-UV-ESI-TOFMS was performed to identify and quantify the polyphenols present on GSE. The HPLC system consisted of Agilent 1200RR chromatograph equipment. A Luna HST 2.5 μm (10 cm × 2.0 mm) column (Phenomenex) was used. The mobile phase consisted of 0.1% formic acid (A) and acetonitrile (B) with the following gradient (t (min), % B): (0, 0), (0.5, 0), (10, 50), (12, 50), (25, 95), (28, 95), (28.5, 0), and (35, 0). The flow rate was 400 μL/min, with no split before the MS. The oven temperature was 50 °C, and the automatic injection system temperature was 10 °C. The injection volume was 10 μL. This HPLC system was coupled to a QSTAR Elite (ABSciex) mass spectrometer fitted with a TurboIon spray source working in negative ionization mode with the following TOFMS conditions: full scan analysis from m/z 100 to 600 and product ion scan m/z 449.

HPLC-UV-ESI-FTMS was performed to identify and quantify the polyphenols present on CE. The HPLC system consisted of Ulltimate 3000 (Dionex) chromatograph equipment. A Kinetex EVO C18 1.7 μm (10 cm × 2.0 mm) column (Phenomenex) was used. The mobile phase consisted of 0.1% formic acid (A) and acetonitrile 0.1% formic acid (B) with the following gradient (t (min), % B): (0, 0), (0.5, 0), (5, 10), (8, 90), (15, 20), (17, 50), (17.5, 50), (18, 95), (19, 95), (19.5, 0), and (22,0). The flow rate was 500 μL/min, with no split before the MS. The oven temperature was 50 °C, and the automatic injection system temperature was 4 °C. The injection volume was 2 μL. This HPLC system was coupled to an LTQ-Orbitrap Velos (Thermo) mass spectrometer fitted with an electrospray source working in negative ionization mode with the following MS conditions: full scan analysis from m/z 100 to 2000 at 30,000 resolution using FTMS.

### Animals

Adult female ICR-CD1 mice (20–30 g) were purchased from Janvier Laboratories (Le-Genest-SaintIsle, France). All animals were housed in groups of 4–5 in standard Marcolon cages (28 × 28 × 15 cm) with wood shaving bedding at 21 ± 1 °C and 40–60% humidity under a 12:12-h light–dark cycle and fed ad libitum with a standard diet of mouse pellets (TEKLAD 2014, Harlan Interfauna Ibérica, Sant Feliu de Codines, Catalonia, Spain). Cages were changed twice weekly. All mice were allowed to acclimatize for at least 1 h to the facility rooms before any functional, behavioral, or surgical procedures, which were all conducted during the light cycle. Sentinel mice were routinely tested for pathogens, and facilities remained pathogen free during the whole experimental period. The number of mice used in this study in all procedures was maintained at a minimum, working with experimental groups consisting of 6 to 12 mice. The animal sample size was calculated using GRANMO (Version 7.12 April 2012) and based on the ethical limits exposed by the Animal Ethics Committee.

All experimental procedures and animal husbandry were conducted following the ARRIVE 2.0 guidelines and performed according to the ethical principles of the IASP for the evaluation of pain in conscious animals^26^ and the European Parliament and the Council Directive of 22 September 2010 (2010/63/EU), along with the approval Ethical Committee on Animal Experimentation (CEEA) of the University of Barcelona and the Department of Agriculture, Livestock, Fisheries, Food and Natural Environment of the Generalitat de Catalunya, Government of Catalonia (DAAM number 9918-P3). All experiments were carried out at the animal experimentation unit of the Bellvitge campus (University of Barcelona).

### Surgical procedure and treatments

Animals were anesthetized with sodium pentobarbital (50 mg/kg, i.p.) and placed prone on a heating pad to maintain constant body temperature levels. After back shaving and disinfection with povidone iodide, T8–T9 thoracic spinal cord segments were exposed via dorsal laminectomy, and using the contusion weight-drop technique, two grams of weight was dropped from 25 mm high onto the metallic stage located over the exposed spinal cord (centered in the midline), to induce mild spinal cord injury^17,27,28^. Following this procedure, the wound was closed, and the animals were allowed to recover in warmed cages with access to food and water. After the surgical procedure, animals also received 0.5 mL saline solution (i.p.) to restore an eventual volemic deficit. In sham animals, the spinal cord was exposed as described above but not contusioned, and they underwent the same recovery procedures.

Thirty minutes after spinal cord injury and daily during the first week postinjury, different sets of spinal cord injury (SCI) animals received intraperitoneal administration of (i) grape stalk extract (GSE) at doses of 10, 15 and 20 mg/kg or (ii) coffee extract (CE) at doses of 10 and 15 mg/kg. Another set of animals also received saline solution, the dilution vehicle of GSE and CE. The experimental groups of the present study were GSE10 (SCI-mice treated with GSE at 10 mg/kg), GSE15 (SCI-mice treated with GSE at 15 mg/kg), GSE20 (SCI-mice treated with GSE at 20 mg/kg), CE10 (SCI-mice treated with CE at 10 mg/kg), CE15 (SCI-mice treated with CE at 15 mg/kg), saline (SCI-mice treated with saline solution at a volume equivalent to that injected in the animals with the dose of 15 mg/kg) and sham (mice with dorsal laminectomy without spinal cord contusion and without treatment).

### Functional evaluation

#### Locomotor activity

Locomotor activity was evaluated using a circular open field (70 cm diameter × 24 cm wall height), where each animal was allowed to move freely for 5 min. Two independent examiners observed the hindlimb movements of the mouse and scored the locomotor function according to the Basso Mouse Scale for locomotion (BMS)^29^. The final score of each animal was the mean value of both examiners. The BMS ranges from 0 (no hindlimb movement) to 9 (normal movement-coordinated gait). The objective of evaluating this test is to determine the state of locomotion of the animals after the contusion of the spinal cord and to verify that all of them have scores on the BMS scale higher than 5–6 points, which means that they maintain the ability to move and/or voluntarily remove the hind legs. This test was evaluated before inducing spinal cord injury and at 7, 14, and 21 days postinjury (dpi).

#### Nociceptive test: mechanical allodynia

Mechanical allodynia was evaluated by assessing 50% withdrawal thresholds using a set of von Frey monofilaments (bending force range 0.04–2 g) following the up-down paradigm^27,28^. Animals were placed in plastic tube test chambers with a metal mesh floor that allowed full access to the plantar surface of the hind paws. Behavioral accommodation was allowed for approximately 1 h until cage exploration and major grooming activities ceased. Then, von Frey monofilaments were perpendicularly applied to the plantar surface with sufficient force to cause slight buckling against the paw. First, the 0.4 g filament was applied, and then, the strength of the filament was decreased when the mouse responded or increased when it did not respond. Clear paw withdrawal, shaking or licking were considered to be a response. This up-down procedure was limited to four assessments after the first response. Each filament was applied for 2 s with interstimulus intervals of 5–10 s. Both paws were evaluated since the SCI model results in bilateral injury, and it is not possible to use the contralateral paw as a natural intraindividual control. The mechanical threshold that produced 50% of responses was calculated using the Dixon formula^30^: 50% paw withdrawal threshold (g) = [(10(Xf + κδ)/10,000)], where Xf is the value (in logarithmic units) of the final von Frey filament used, κ is a fixed tabular value for the pattern of positive/negative responses and δ is the mean difference (in logarithmic units) between stimuli. This test was evaluated before inducing SCI and at 7, 14, and 21 dpi and after the evaluation of locomotor activity.

#### Nociceptive test: thermal hyperalgesia

Thermal hyperalgesia was assessed by measuring the hind paw withdrawal latency in response to a thermal stimulus. Plantar tests were performed according to the Hargreaves method^17,27,28,31^ using a plantar test analgesimeter (#37370; Ugo Basile, Comerio, Italy). Mice were placed into plastic test enclosures with an elevated glass floor and allowed to acclimate for approximately 1 h until cage exploration and major grooming activities ceased. Then, the light of a projection lamp (100 W) was focused onto the plantar surface of the hind paw with a time limit of 30 s to avoid skin damage. Withdrawal latency was automatically recorded by a time-meter coupled to infrared detectors directed to the plantar surface of the paw. The sum of the mean withdrawal latencies for both hind paws was determined from the average of three separate trials conducted at 5-min intervals. This test was also evaluated before inducing spinal cord injury and at 7, 14 and 21 dpi and after mechanical allodynia evaluation.

### Tissue sample collection

At 21 dpi and after functional evaluation, animals were anaesthetized with sodium pentobarbital (90 mg/kg; i.p.) Then, blood was extracted through the insertion of an intracardiac needle. Subsequently, the obtained blood was centrifuged for 15 min at 4000 rpm to obtain the serum, which was frozen immediately in dry ice and stored at − 80 °C until analysis.

On the one hand, half of the animals in each experimental group were perfused intraventricularly with 4% paraformaldehyde solution in phosphate-buffered saline (PBS, 10 mM sodium phosphate buffer, pH 7.4). Afterwards, spinal cord, brainstem and brain tissues were carefully removed. Spinal cord samples were immersed in a Zamboni fixing solution (4% paraformaldehyde and 0.3% picric acid in PBS^32^). The spinal cord samples were conserved in Zamboni solution at least 14 days after extraction and then were preserved in 30% sucrose solution in PBS. These samples were stored at 4 °C until histological analysis by immunohistochemical techniques. On the other hand, the entire brain, including the brainstem, was removed from each mouse and postfixed in 4% paraformaldehyde at 4 °C until histological analysis. In the other half of the animals in each experimental group, the spinal cord was exposed by a dorsal laminectomy and was then meticulously extracted and stored at − 80 °C. In addition, the entire brain was removed from the cranium of each mouse and stored at − 80 °C. All these samples were used for molecular biology studies.

### Histological analysis

#### Spinal cord samples

For the immunohistochemical analysis, T7-T10 spinal cord segments were embedded in Tissue Freezing Medium (0201-08926, Leica, Barcelona) and cut transversely with a cryostat (CM1520, Leica, Barcelona) into 60 µm thick sections that were collected in six-well porcelain plates. Concretely, the T7-T10 spinal cord block was cut in half at the level of T8, generating a rostral (T7-T8) and a caudal (T9-T10) block. Starting from T8, transverse sections of the spinal cord were made. First, sections were washed 2 times for 10 min with saline phosphate buffer (PBS, 0.1 M, pH = 7.4) and 2 times for 10 min with 0.1 M PBS/0.3% Triton (PBS-Triton). Next, tissue sections were blocked with 1% bovine fetal serum in PBS-Triton (PBS-Triton-FCS) for 1 h and were then incubated with rabbit anti-GFAP (Glial fibrillary acidic protein; 1:200, ab7260, Abcam) or rabbit anti-IBA1 (Ionized calcium-Binding Adaptor molecule 1; 1:200, 019-19741, Fujifilm Wako) for 48 h at 4 °C in a humidified chamber to avoid tissue drying out. As a specificity control, some spinal cord sections were incubated without primary antibody. The sections were washed three times for 10 min with PBS-Triton and then incubated overnight at 4 °C in a humidified chamber with AffiniPure goat anti-rabbit IgG conjugated with cyanine 3 (Cy3) (1:200, Ref # 111-165-144, Jackson ImmunoResearch, USA). Finally, two 10-min washes were again performed with PBS-Triton and one 10-min wash with PBS, and the samples were mounted on previously gelatinized slides. Once mounted, slides were dehydrated through immersion in increasing concentrations of ethanol baths (70%, 96% and 100%) and were covered with cover glass fixed with DPX mounting media (1.01979.0500, Merck, Germany)^17^.

Histological sections were observed with an epifluorescence microscope (Leica DMR-XA; Leica Microsystems) attached to a digital camera (FMVU-13S2C-CS; Point Gray Research, Canada) used to capture the images (×200). The resulting images were analyzed with the free software ImageJ (Image Processing and Analysis in Java, National Institute of Health, NIH, USA). The ×200 immunolabeled images for GFAP and Iba1 were taken preferentially in the gray matter of the dorsal horn of the spinal cord, although in some cases white matter tracts were also taken very partially. For each animal in the present study, a minimum of eight immunolabeled histological sections were analyzed. Imaging of the sections immunolabeled for IBA1 was performed for reactive and nonreactive microglial cells and was expressed as a percentage of two phenotypes. The percentage of reactive microglial cells was considered an index of the degree of microgliosis. Regarding GFAP immunolabeling, the area immunopositive for GFAP was measured as a percentage of immunoreactivity^17^. In addition, whole spinal cord coronal sections images were captured to observe the preservation of ventrolateral funiculus in all experimental groups. Whole sections of the spinal cord were taken at ×50.

#### Brain and brainstem samples: supraspinal structures

Serial PAG coronal sections (12 µm) from the central part of the superior colliculi to the upper edge of the inferior colliculi corresponding to PAG between 6.72 and 8.04 mm from the bregma^33^ were cut (Leica 1800 cryostat; Leica Microsystems, Wetzlar, Germany). For ACC, serial coronal sections (12 µm) through the prefrontal cortex ACC between 2.2 and 4.2 mm from the bregma^33^ were also prepared.

The sections were collected on gelatin-coated microscopic slides, air-dried, and processed for immunohistochemical staining. First, the sections were washed with PBS containing 0.05% Tween 20 (PBS-TW20) and 1% bovine serum albumin for 10 min and then treated with 3% normal donkey serum in PBS-TW20 for 30 min. Then, the sections were incubated with rabbit anti-GFAP (1:250; DAKO) or rabbit anti-IBA1 (1:100; Wako) antibodies in a humid chamber at room temperature for 12 h. The binding of primary antibodies was visualized by secondary antibodies (FITC- or TRITC-conjugated, affinity purified goat anti-rabbit; 1:100; Jackson) for 90 min at room temperature. To detect the M2 phenotype subtype of microglial cells, some PAG sections were double immunostained with mouse anti-OX42 (1:100; Santa Cruz) and rabbit anti-CD206 (1:100; Abcam) primary antibodies and developed with purified goat anti-mouse and anti-rabbit FITC- and TRITC-conjugated secondary antibodies, respectively. Immunostained sections were rinsed, stained with Hoechst 33342 to detect the positions of the cell nuclei, and mounted in Vectashield aqueous mounting medium (Vector Laboratories Inc., Burlingame, CA). The control sections were incubated with omission of the primary antibody and displayed no immunostaining.

PAG and ACC sections were analyzed using a Nikon Eclipse NI-E epifluorescence microscope equipped with a Nikon DS-Ri1 camera driven by NIS-elements software (Nikon, Prague, Czech Republic). The areas immunopositive for GFAP or IBA1 were detected by a thresholding technique after subtraction of the background. The areas of immunostaining for GFAP or IBA1 were related to the areas of interest and expressed as the proportion of relative area ± SEM. Quantification of OX42-immunostained and OX42/CD206 double-immunostained microglial cells was carried out using a Leica TCS SP5 confocal microscope at 20× magnification (Leica HC APO L 20×/0.50 W objective). Stacks of confocal images across 6 randomly selected regions of interest were collected from 4 whole sections through PAG. A proportion of OX42/CD206 double immunostained M2 subtype microglial cells to all OX42-immunopositive cells was counted from the results examined by 2 investigators blinded to the tissue source.

### Molecular biology analysis: western blot

#### Spinal cord samples

Spinal cord tissue was homogenized in modified RIPA buffer (50 mM Tris–HCl pH 7.5, 1% Triton X‐100, 0.5% sodium deoxycholate, 0.2% SDS, 100 mM NaCl, 1 mM EDTA, 2 mM PMSF, 1 µg/μL aprotinin, 1 µg/μL leupeptin, and 2 mM sodium orthovanadate) and was then centrifuged at 18,000*g* at 4 °C for 30 min. The protein concentration from the obtained supernatant was determined by Protein Assay DCTM (Bio‐Rad). The samples were then stored at − 80 °C until use.

Samples containing equal amounts of protein (10–20 µg) were mixed with 2× Laemmli sample buffer (S3401, Sigma-Aldrich) and boiled at 95 °C for 10 min. Samples were fractioned by 10–15% (w/v) SDS-PAGE gels and transferred onto a nitrocellulose membrane and then blocked with either 5% nonfat dry milk or bovine serum albumin (BSA) in Tris-0.1% Tween 20-buffered saline (T-TBS) for 2 h at room temperature.

Membranes were incubated with the following primary antibodies overnight at 4 °C: rabbit anti-IBA1 (1:700, 0019-19741, FUJIFILM Wako Chemicals), rabbit anti-GFAP (1:800, SAB4300647, Sigma-Aldrich), rabbit anti-MCP1/CCL2 (1:1000, ab25124, Abcam), rabbit anti-CCR2 (1:1000, ab203128, Abcam), rabbit anti-CX3CL1 (1:1000, ab25088, Abcam), rabbit anti-CX3CR1 (1:1000, ab8021, Abcam), rabbit anti-extracellular signal-regulated kinases (total ERK ½) (1:1000), and diphosphorylated ERKs (pERK ½) (1:1000). Rabbit anti-GAPDH antibody (1:10,000, G9545, Sigma-Aldrich) was used as a loading control.

The immunoblots were washed three times for 10 min with T-TBS and then incubated for 1:30 h at room temperature with horseradish peroxidase-conjugated goat antirabbit IgG (1/50,000, AP132P, Sigma-Aldrich) and revealed by chemiluminescence Clarity Western ECL Substrate (170-5061, Bio-Rad). Band pixel intensities were quantified by Gel‐Pro Analyzer software (Media Cybernetics, USA) and normalized to the corresponding GAPDH intensity. pERK ½ was normalized to total ERK and subsequently normalized to GAPDH immunoreactivity intensity. In the supplementary material are available the original scanned/revealed full blots. Note that considering that some spinal cord sample blots have been manually developed with a film, without a developer machine and it is not possible to visualize the membrane on the developed films of GFAP, IBA1, CCR2, CX3CR1 and pERK/ERK. The blots were cut prior to hybridization with the antibody of interest and GAPDH, the loading control and consequently not all blot images present exactly the same length.

#### Supraspinal structures

To obtain the PAG area, a 2 mm thick coronal slice of the mesencephalon was carefully removed at the position from the central part of the superior colliculi to the upper edge of the inferior colliculi corresponding to PAG between 6.72 mm and 8.04 mm from the bregma. For ACC, a 2 mm thick coronal slice of the prefrontal cortex was carefully removed at the position between 2.2 mm and 4.2 mm from the bregma^33^.

The PAG and ACC tissue samples of individual animals were homogenized in RIPA buffer (Abcam) containing protease inhibitors (LaRoche, Switzerland) and were then centrifuged at 15,000*g* at 4 °C for 20 min. The protein concentration from the tissue supernatant was measured by a Nanodrop ND-1000 (Thermo Scientific) and normalized to the same levels.

Each sample, containing 50 µg of protein, was separated by SDS–polyacrylamide gel electrophoresis and transferred onto nitrocellulose membranes. The membranes were then blocked with 1% BSA in PBST (3.2 mM Na_2_HPO_4_, 0.5 mM KH_2_PO_4_, 1.3 mM KCl, 135 mM NaCl, 0.05% Tween 20, pH 7.4) for 1 h at room temperature and incubated with the following primary antibodies overnight at 4 °C: rabbit anti-IBA1 (1:100, FUJIFILM Wako Chemicals), rabbit anti-GFAP (1:10,000, Abcam), rabbit anti-CX3CL1 (1:2000, Abcam), and rabbit anti-CX3CR1 (1:2000, Abcam). Mouse anti-α-tubulin antibody (1:1000, Cell Signaling) was used as a loading control.

Blots were washed in PBST and incubated with peroxidase-conjugated anti-mouse or anti-rabbit secondary antibodies (Sigma, 1:1000) at room temperature for 1 h. Protein bands were visualized using the ECL detection kit (Amersham) on an LAS-3000 chemiluminometer reader (Bouchet Biotech) and analyzed using densitometry image software.

### Biochemical analysis of hepato- and nephrotoxicity

Serum samples were analyzed to study the possible hepatotoxicity and nephrotoxicity produced by the different vegetable extract treatments. To this end, serum levels of alanine aminotransferase (ALT/GPT; # 11533, BioSystems, Barcelona, Spain), aspartate aminotransferase (AST/GOT; #11531, BioSystems, Barcelona, Spain) and urea-BUN (# 11536, BioSystems, Barcelona, Spain) were determined by using commercial assay kits according to the manufacturer’s instructions.

### Statistical analysis

All functional, histological, and biochemical analyses were performed in a blinded manner using a code for each mouse. The results are shown as the mean ± standard deviation of the mean (SEM). The normal distribution of the data was analyzed by Shapiro–Wilk or Kolmogorov–Smirnov tests before further applying parametric or nonparametric statistical analyses. Data that followed a normal distribution were analyzed using repeated measures MANOVA (Wilks’ criterion) and analysis of variance (ANOVA) followed by Duncan’s test, when applicable. Data that did not follow a normal distribution were analyzed using the Friedman statistic test for nonparametric repeated measures and Kruskal–Wallis followed by the Mann–Whitney U test. In all statistical analyses, the α level was set at 0.05 using the statistical package SPSS 25.0 for Windows.

### Ethics approval and consent to participate

All experimental procedures and animal husbandry were performed according to the ethical principles of the I.A.S.P. for the evaluation of pain in conscious animals and the European Parliament and the Council Directive of 22 September 2010 (2010/63/EU), along with the approval Ethical Committee on Animal Experimentation (CEEA) of the University of Barcelona and the Department of Agriculture, Livestock, Fisheries, Food and Natural Environment of the Generalitat de Catalunya, Government of Catalonia (DAAM number 9918-P3). All experiments were carried out at the animal experimentation unit of the Bellvitge campus (University of Barcelona).

## Results

### Polyphenol content and HPLC analysis in the GSE and CE extracts

Several batches of both GSE and CE were obtained to perform the scheduled experimental procedures. According to the Folin-Ciocalteu assay, the total polyphenolic content of GSE and CE batches are indicated in Table 1. The total polyphenolic content obtained after the extraction procedure was approximately 1100 mg GAE/L on average for GSE and 2200 mg GAE/L on average for CE. These polyphenol concentrations allowed the preparation of a suitable volume of administration of experimental doses.

**Table 1:** The total polyphenolic content in GSE and CE was calculated according to the Folin-Ciocalteu method.

Batch number	Polyphenol content in GSE (mg GAE/L)	Polyphenol content in CE (mg GAE/L)
1	960 ± 33	1985 ± 124
2	1014 ± 39	2186 ± 137
3	1104 ± 60	2456 ± 153
4	1285 ± 82	2480 ± 130

Polyphenol content is expressed as milligrams of gallic acid equivalents (GAE) per liter of extract. All values are presented as the mean ± SD (n = 6).

**Figure 1:**
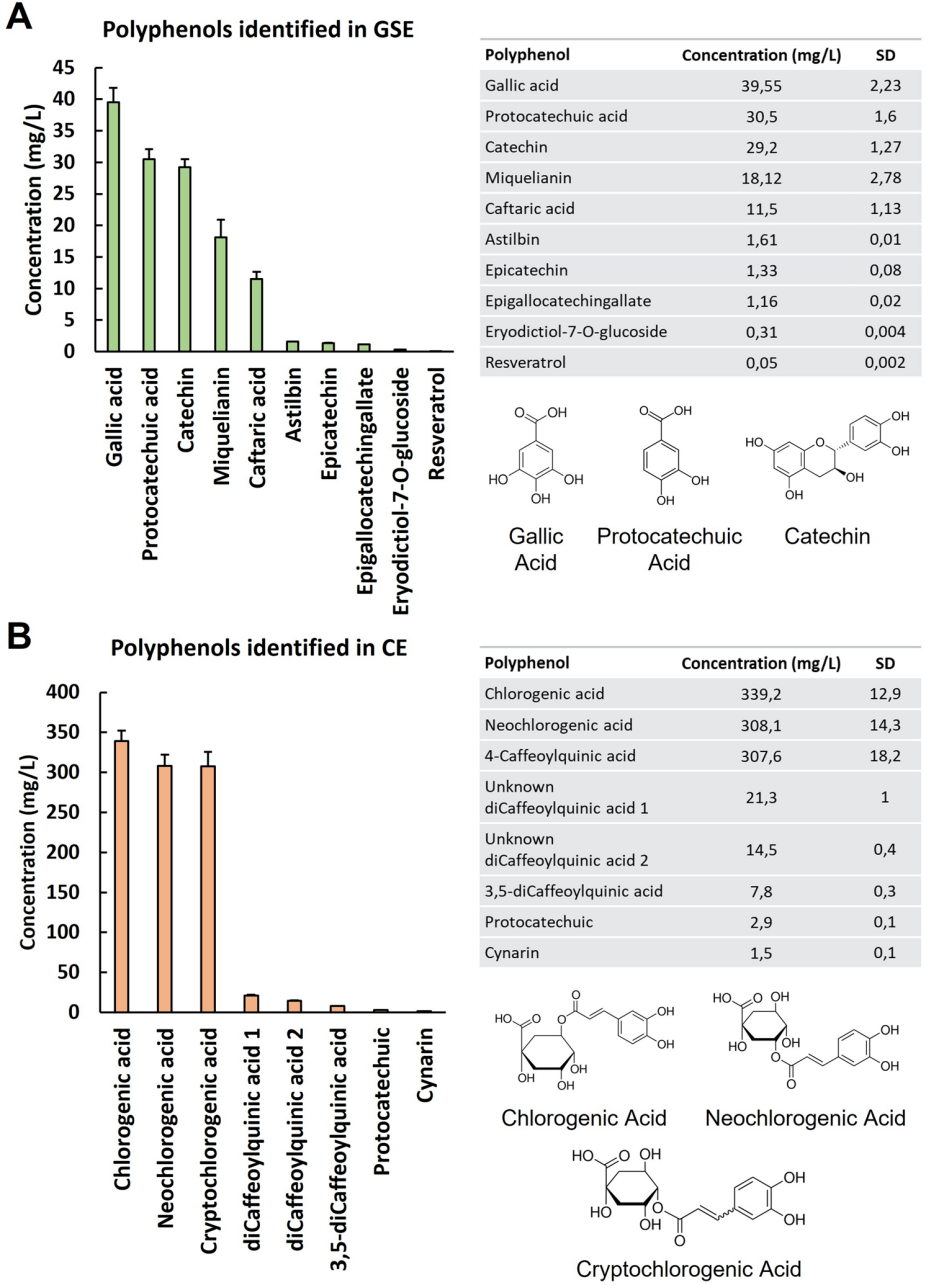
Quantification of phenolic compounds in GSE (**A**) and CE (**B**) by HPLC**.** The results are presented as a histogram and values data table. Quantification of each polyphenol identified in both extracts was performed in triplicate, and all values are presented as the mean ± SD (n = 3). The molecular structures of the major compounds in both extracts are also presented.

Further HPLC analysis revealed that the grape stalk extract (GSE) used in the present study showed gallic acid, protocatechuic acid and catechin as major polyphenolic compounds. Additionally, other polyphenols present at lower concentrations were miquelianin, caftaric acid, astilbin, epicatechin, epigallocatechin-gallate, erydictol-7-O-glucoside and resveratrol (Fig. 1A). For the coffee extract (CE) used in this work, HPLC analysis showed that chlorogenic acid, neochlorogenic acid and cryptochlorogenic acid were the major polyphenols in this extract. Other polyphenolic compounds also observed in CE at lower concentrations were 3,5-di-caffeoylquinic acid, protocatechuic acid, cynarin and two types of dicaffeoylquinic acid (Fig. 1B). The concentrations of the polyphenols identified in both extracts are indicated in Fig. 1.

### GSE and CE administration did not cause neither hepato- nor nephrotoxicity in mice subjected to a spinal cord contusion

**Figure 2:**
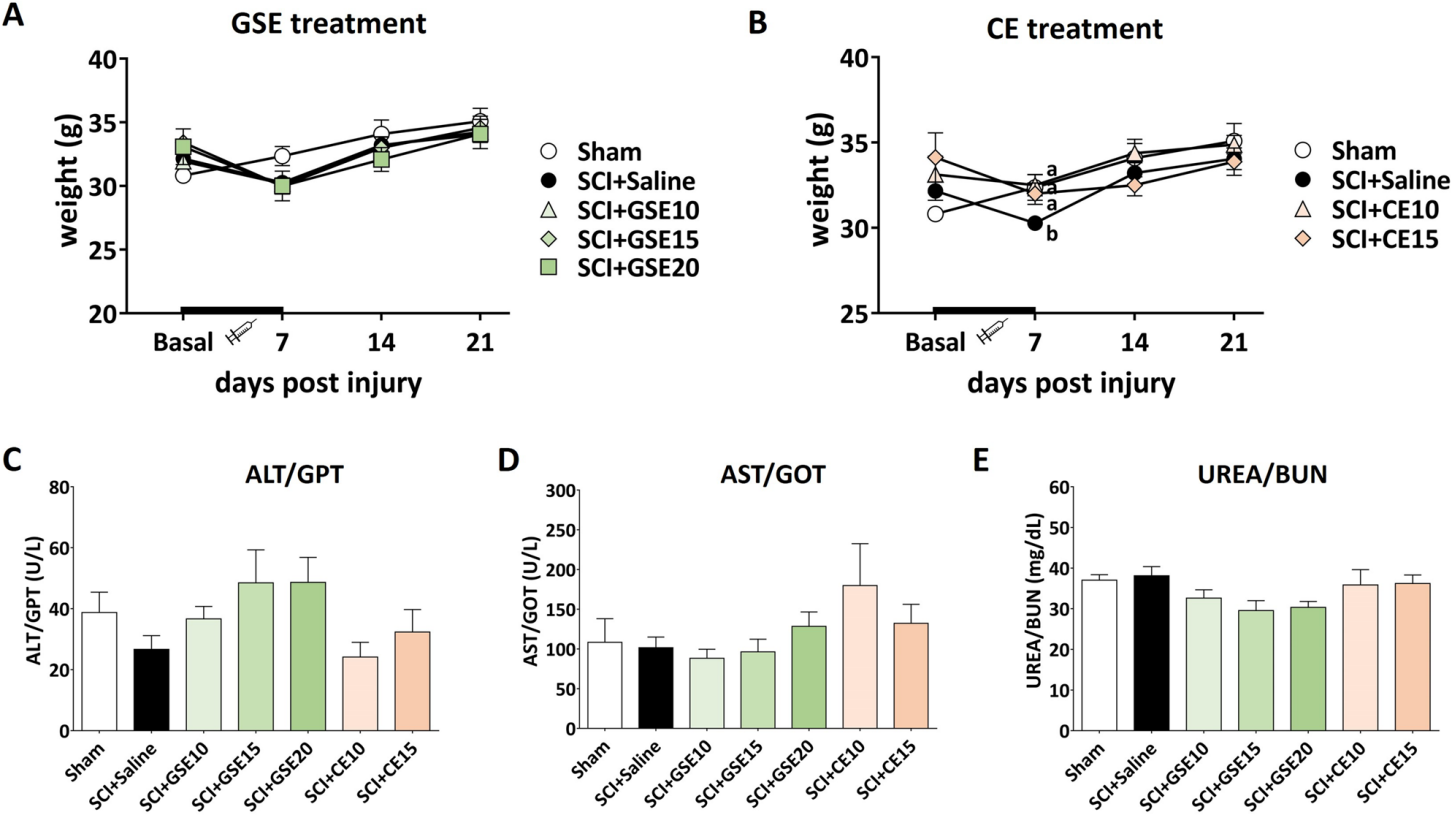
Mouse weight control during the injury acute phase of SCI after preventive (**A**) GSE and (**B**) CE treatment and biomarkers quantification of (**C**,**D**) hepatotoxicity and (**E**) nephrotoxicity in the serum of each experimental group at the end of the experimental period. The results are represented as the mean ± SEM. The treatment administration week (basal to 7 dpi) is highlighted with a thick black line (**A**,**B**). a–b: Groups not sharing a letter showed significant differences, p < 0.05. Experimental groups (A-B): Sham (n = 11), SCI + Saline (n = 18), SCI + GSE10 (n = 16), SCI + GSE15 (n = 14), SCI + GSE20 (n = 12), SCI + CE10 (n = 8), SCI + CE15 (n = 8). Experimental groups (C-E): Sham (ALT/GTP n = 6; AST/GOT n = 6; UREA n = 6), SCI + Saline (ALT/GTP n = 6; AST/GOT n = 6; UREA n = 6), SCI + GSE10 (ALT/GTP n = 6; AST/GOT n = 5; UREA n = 6), SCI + GSE15 (ALT/GTP n = 5; AST/GOT n = 6; UREA n = 7), SCI + GSE20 (ALT/GTP n = 5; AST/GOT n = 5; UREA n = 5), SCI + CE10 (ALT/GTP n = 5; AST/GOT n = 5; UREA n = 6), SCI + CE15 (ALT/GTP n = 6; AST/GOT n = 6; UREA n = 6).

Throughout the study and following a protocol animal welfare supervision based on Morton D.B. and Griffiths P.H. guidelines^34^, the general aspect of the animals was normal, and changes in coat and skin, vibrissae of nose, nasal secretions, signs of autotomy or aggressiveness were not detected in any experimental group of mice. Animals treated with either GSE or CE showed no significant difference in body weight compared to those in the control group Sham (Fig. 2A,B). Regarding the pharmacological safety of the treatments, the administration of GSE and CE did not alter the biomarkers of hepatotoxicity (ALT/GPT and AST/GOT) and nephrotoxicity (UREA/BUN) in the animals’ serum compared to non-administered animals (all p’s > 0.05) (Fig. 2C–E). These results show that no systemic toxicity may be associated with the polyphenolic extracts.

### Both GSE and CE treatments alleviate the development of reflexive pain responses in SCI mice.

**Figure 3:**
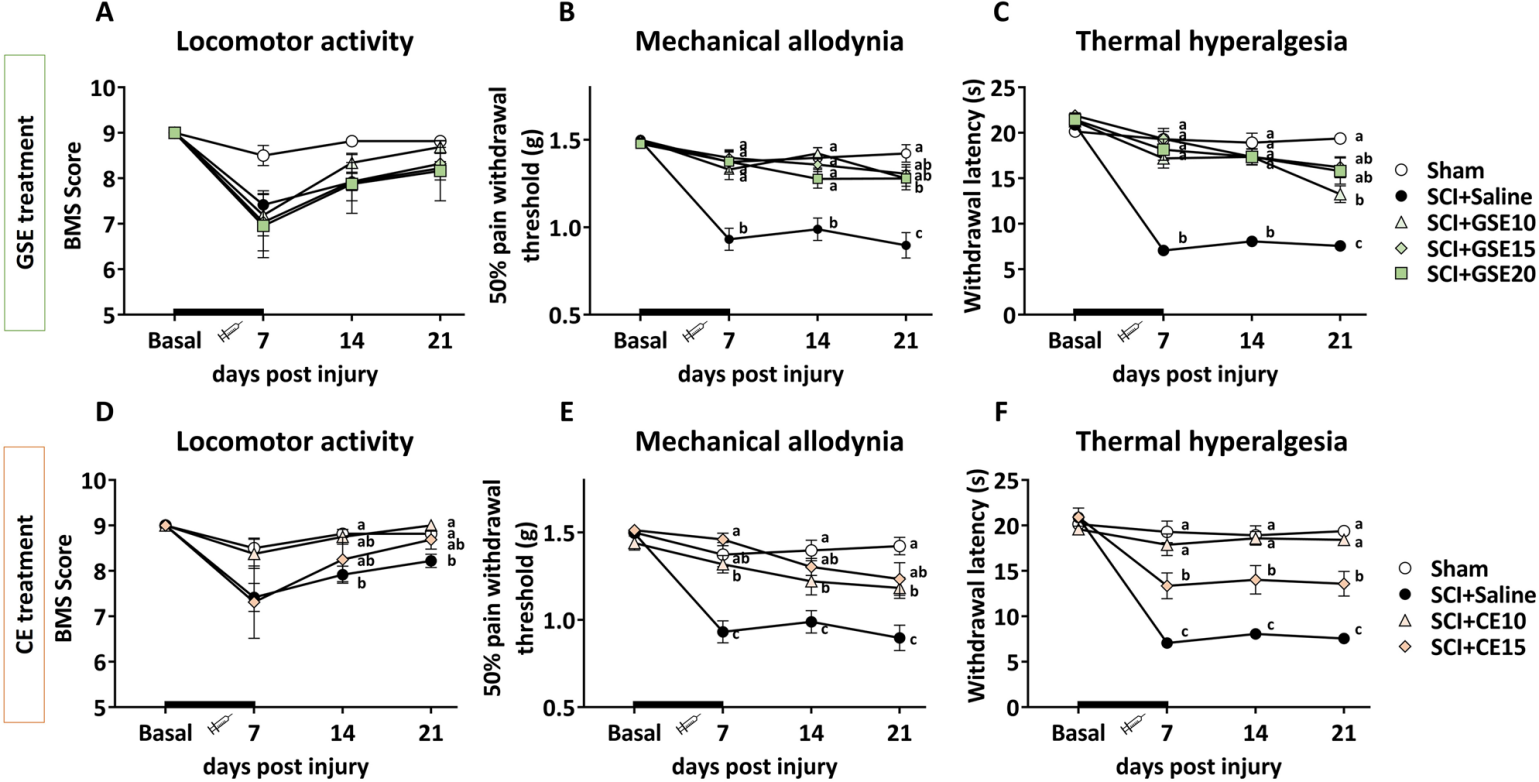
Time-course assessment of locomotor activity (**A**,**D**), mechanical allodynia (**B**,**E**) and thermal hyperalgesia (**C**,**F**) during the injury acute phase of SCI after preventive GSE and CE treatments. Each point and vertical line represent the mean ± SEM. The treatment administration week (basal to 7 dpi) is highlighted with a thick black line. a–c: Groups not sharing a letter showed significant differences, p < 0.05. Experimental groups: Sham (n = 11), SCI + Saline (n = 18), SCI + GSE10 (n = 16), SCI + GSE15 (n = 14), SCI + GSE20 (n = 12), SCI + CE10 (n = 8), SCI + CE15 (n = 8).

Although SCI, whether treated with GSE (10, 15 and 20 mg/kg) or CE (10 and 15 mg/kg), caused a slight decrease in motor response at 7 days postinjury compared to Sham animals, all animals showed a motor function recovery to the levels observed in Sham animals up to the end of the follow-up (Fig. 3A,D). Throughout the whole experimental period, most of the animals in the different experimental groups presented BMS scores greater than 6, referring to altered paw position but not to altered horizontal locomotion. Therefore, the induced contusion did not cause paralysis, and all mouse groups were able to move freely without major locomotor interferences on functional evaluations.

Regarding reflexive pain responses, SCI animals treated with vehicle (saline) showed significant mechanical allodynia development compared with sham animals at all experimental time points (7, 14 and 21 dpo; all p < 0.05). In contrast, GSE and CE treatments significantly alleviated this reflexive-pain response development up to the end of the experimental period (all p’s < 0.05). Concretely, at 21 days postinjury, both GSE10 and GSE15 mice showed similar values to the Sham group at the withdrawal threshold to mechanical stimulation (all p’s > 0.05), while GSE20 animals showed significantly lower withdrawal threshold values in comparison with the Sham group (p < 0.05), suggesting that the 10 and 15 mg/kg doses of GSE alleviate mechanical allodynia development more than the 20 mg/kg dose (Fig. 3B). Regarding CE treatment, both CE10 and CE15 showed similar effects on the withdrawal threshold to mechanical stimulation (p = 0.382). However, the CE10 showed significant differences with respect to the Sham group (p < 0.01), while the CE15 did not show such significant differences (p = 0.062), suggesting that the higher dose relieved mechanical allodynia more than the lower dose of CE (Fig. 3E).

On the other hand, during all time-points of functional assessment, SCI mice without treatment (Saline groups) showed significant thermal hyperalgesia development when compared to Sham (all p’s < 0.05). Specifically, at 21 dpi, GSE15 and GSE20 animals showed similar values of withdrawal latency to thermal stimulation compared to Sham animals (p > 0.05). In contrast, GSE10 mice showed significant differences from the Sham group (p < 0.05), suggesting that GSE doses of 15 and 20 mg/kg were more effective than the 10 mg/kg dose in alleviating thermal hyperalgesia development (Fig. 3C). Regarding CE treatment, the 10 mg/kg dose was significantly more effective than the 15 mg/kg dose in alleviating thermal hyperalgesia development, as the Sham and CE10 groups did not differ statistically (p = 0.362), while Sham and CE15 groups did (p < 0.05) (Fig. 3F).

### GSE and CE treatments reduce spinal cord injury-induced astrogliosis and microgliosis

**Figure 4:**
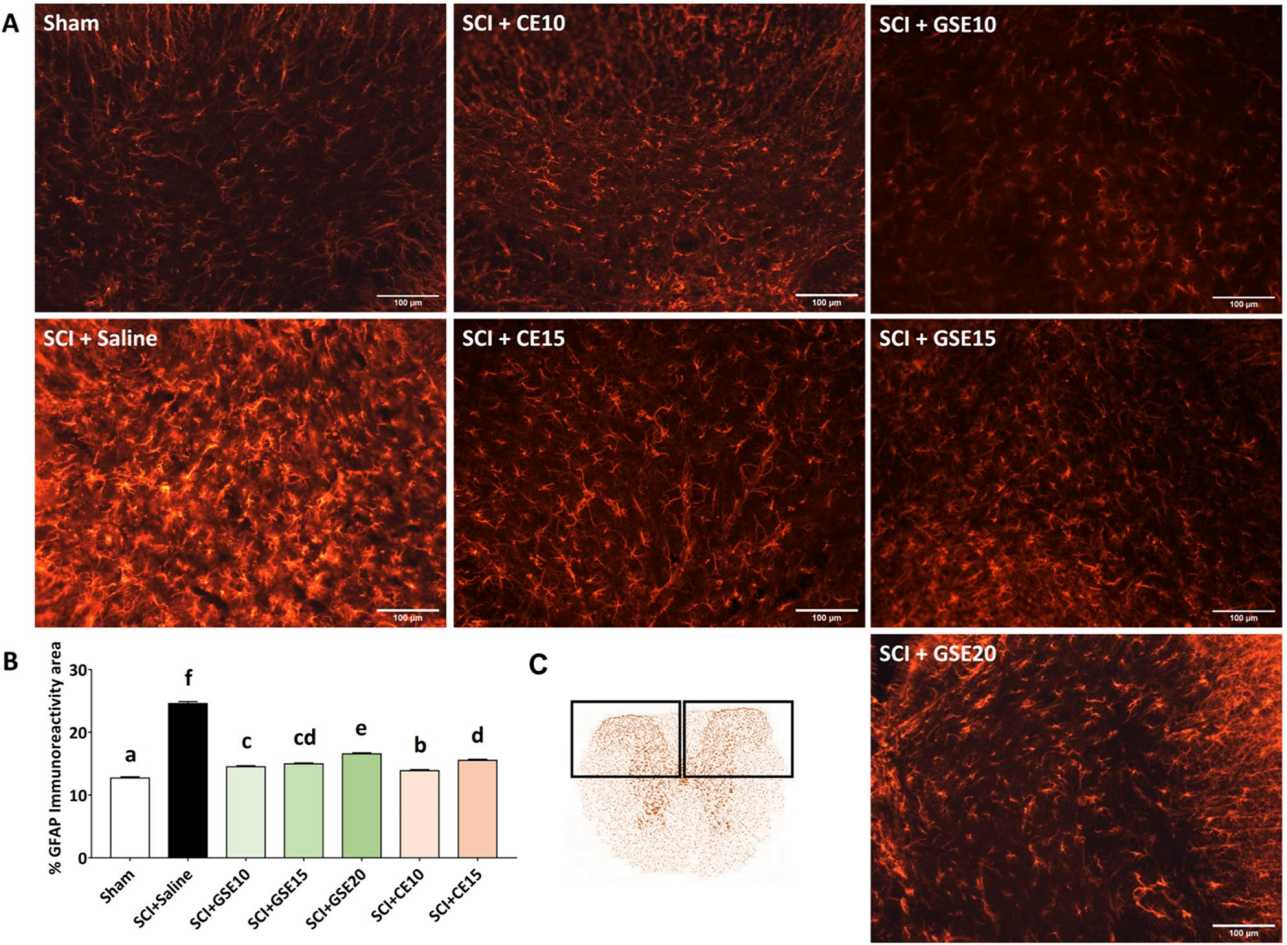
Effects of GSE and CE treatment on the spinal GFAP immunoreactivity area in SCI mice at the end of the experimental period**.** (**A**) Representative histological images of the spinal cord immunostained against GFAP of each group (scale bar 100 μm). (**B**) Histograms representing the percentage of dorsal horn GFAP immunoreactivity. (**C**) Schematic representation of spinal cord section showing where images capture and immunohistochemical analysis were performed. Data are expressed as the mean ± SEM. a–f: Groups not sharing a letter showed significant differences, p < 0.05. Experimental groups: Sham (n = 4; slices = 60), SCI + Saline (n = 4; slices = 62), SCI + GSE10 (n = 4; slices = 68), SCI + GSE15 (n = 5; slices = 84), SCI + GSE20 (n = 5; slices = 54), SCI + CE10 (n = 5; slices = 58), SCI + CE15 (n = 4; slices = 67).

In SCI animals treated with vehicle (Saline), a significant (all p’s < 0.05) increase in astrogliosis was observed compared to control animals (Sham group) at the end of the experimental period. Histological images showed that in the spinal cord of SCI animals, astrocytes were hypertrophic, with thick cytoplasmic processes and intense GFAP labeling, while astrocytes in the control group (sham) showed thin cytoplasmic processes and were not very GFAP labeled (Fig. 4A). Treatments with either GSE or CE significantly reduced astrogliosis when compared with vehicle-treated animals (Saline; all p’s < 0.05), even though such reduction did not reach Sham data (Fig. 4). Moreover, the whole images of spinal cord sections revealed that the ventrolateral funiculus was preserved on both sides (see Supplementary Fig. S1).

**Figure 5:**
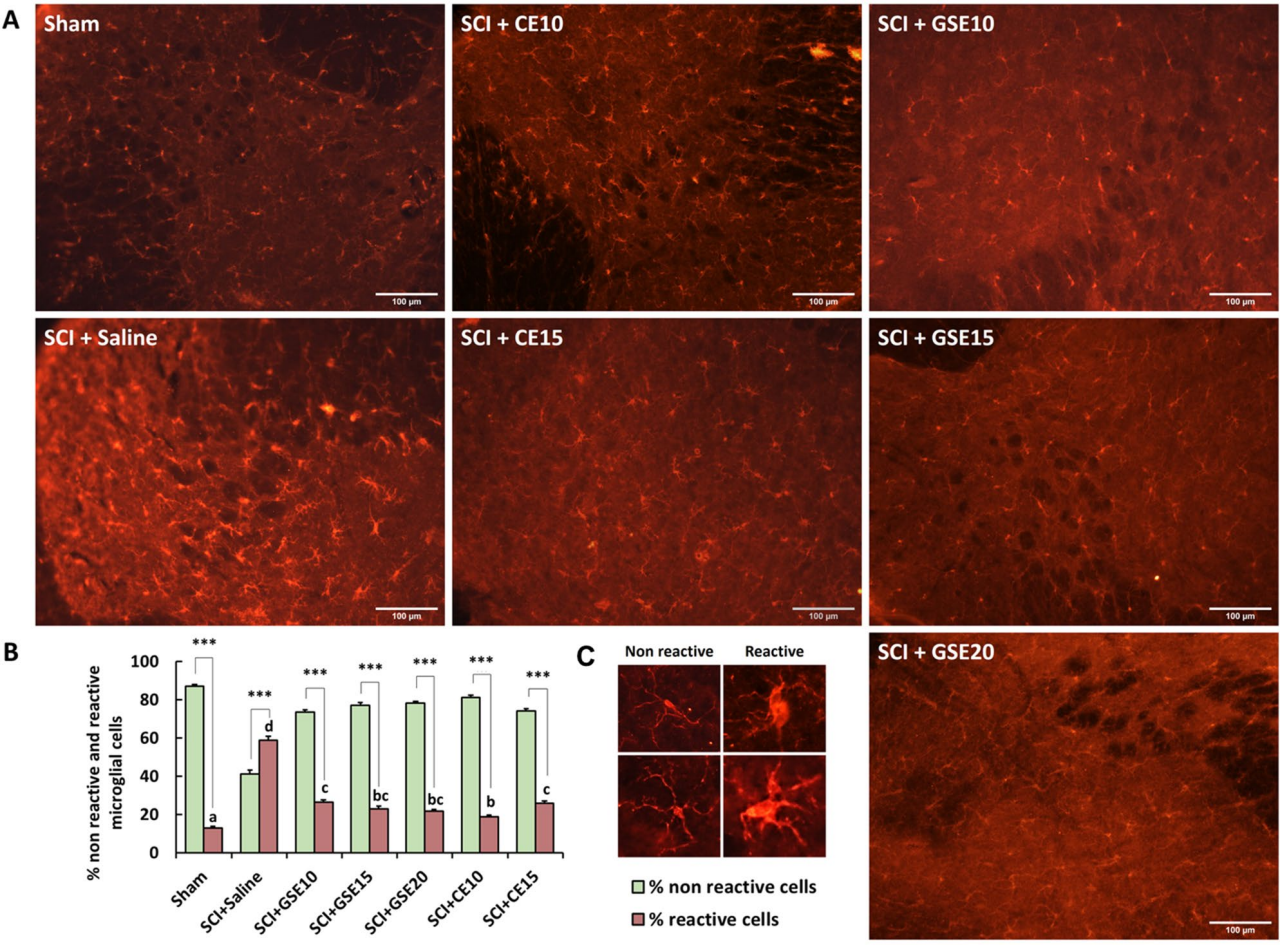
Effects of GSE and CE treatments on spinal IBA1 immunoreactivity in SCI mice at the end of the experimental period. (**A**) Representative histological images of the spinal cord immunostained against IBA1 of each group (scale bar 100 μm). (**B**) Histogram representing the percentage of reactive and nonreactive microglial cells in the spinal dorsal horn. (**C**) Examples of nonreactive and reactive microglial cells. Note that reactive cells have an amoeboid form, a larger nucleus and shorter branching processes than nonreactive cells. Images were captured at the same regions where astrogliosis was analyzed Data are expressed as the mean ± SEM. a–d: Groups not sharing a letter showed significant differences in %reactive cells, p < 0.05. Intragroup significant differences: *** p < 0.001%nonreactive *vs* %reactive. Experimental groups: Sham (n = 6; slices = 53), SCI + Saline (n = 5; slices = 34), SCI + GSE10 (n = 6; slices = 65), SCI + GSE15 (n = 4; slices = 47), SCI + GSE20 (n = 6; slices = 78), SCI + CE10 (n = 5; slices = 67), SCI + CE15 (n = 4; slices = 57).

Regarding spinal microgliosis, SCI animals treated with vehicle (saline) showed a significant (p < 0.001) increase in the percentage of reactive microglial cells compared with the sham group (Fig. 5). Regarding GSE and CE treatments, all doses of both treatments clearly reduced the percentage of reactive cells compared to non-treated SCI animals (p’s < 0.05 vs. SCI) but without reaching the percentage level of the control group (p’s < 0.05 vs. Sham) (Fig. 5). Finally, statistical intragroup differences between reactive and nonreactive microglial cells for all experimental groups were found. Specifically, SCI animals treated with vehicle (saline) showed a significantly (all p’s < 0.01) higher percentage of reactive cells than nonreactive cells. In contrast, animals in the sham group and those treated with GSE and CE showed a higher percentage of nonreactive cells than reactive cells (all p’s < 0.001) (Fig. 5B). In the histological sections (Fig. 5A), it can be observed that nonreactive microglial cells have a small soma from which thin cytoplasmic processes arise. In contrast, reactive microglial cells have an amoeboid morphology, with a large soma and short cytoplasmic branches. IBA1 labeling in reactive microglial cells was more intense than that in nonreactive microglial cells.

**Figure 6:**
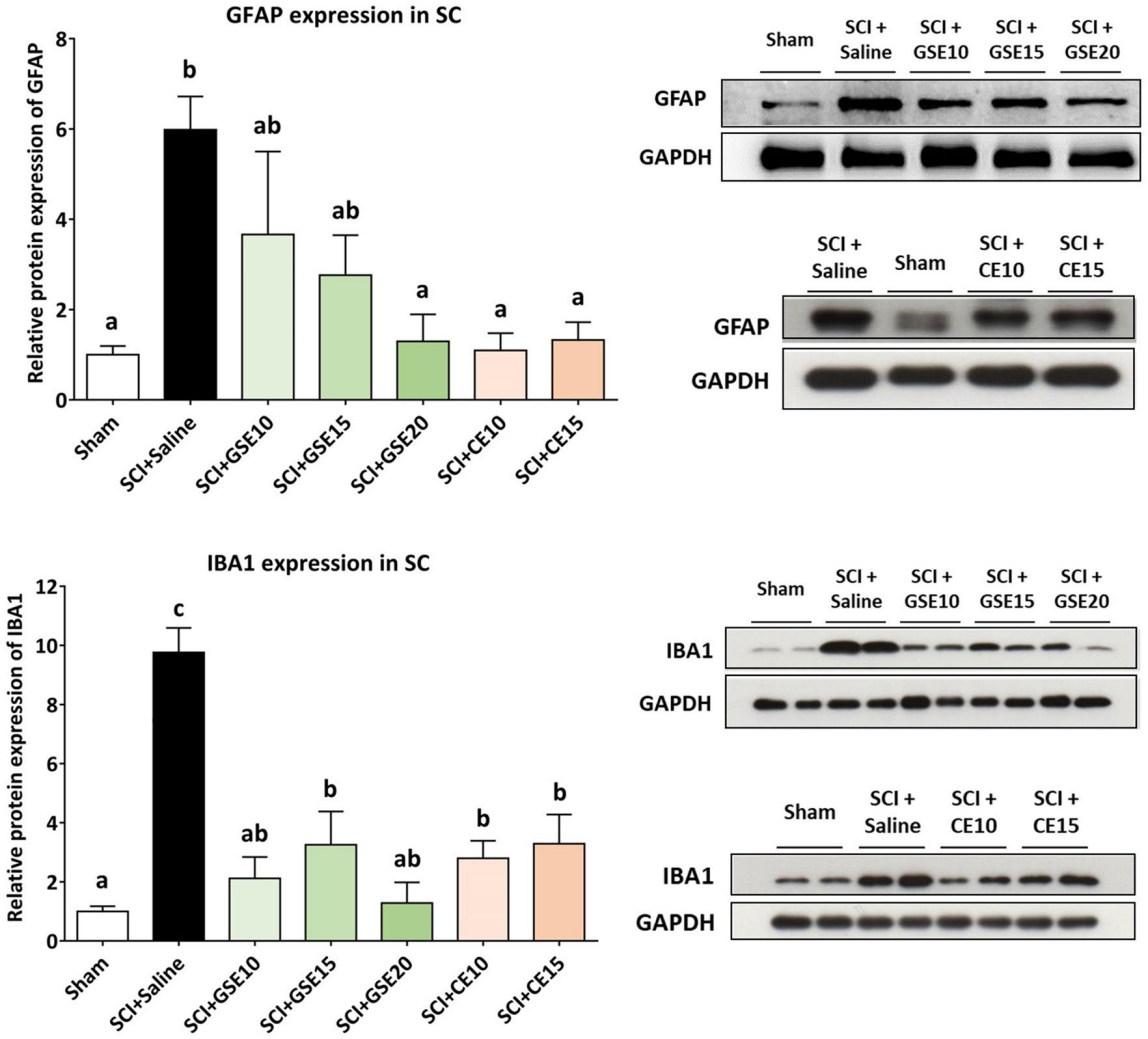
Spinal gliosis (GFAP and IBA1 expression) after preventive GSE and CE treatments in spinal cord-injured mice at the end of the experimental period. Protein expression was normalized to GAPDH. Data are expressed as a relative percentage with respect to the sham group (mean ± SEM). a–c: Groups not sharing a letter showed significant differences, p < 0.05. Experimental groups: GFAP: sham (n = 10), SCI + saline (n = 9), SCI + GSE10 (n = 5), SCI + GSE15 (n = 5), SCI + GSE20 (n = 5), SCI + CE10 (n = 5), SCI + CE15 (n = 5). IBA1: sham (n = 8), SCI + saline (n = 7), SCI + GSE10 (n = 4), SCI + GSE15 (n = 4), SCI + GSE20 (n = 4), SCI + CE10 (n = 4), SCI + CE15 (n = 4). Control images were reused either for illustrative purposes or methodological purposes when several protein levels were assessed in one blot. Full-length blots are presented in Supplementary Fig. S2.

When the expression of GFAP and IBA1 was assessed by molecular biology techniques, it was observed that in animals with SCI and treated with saline solution, the expression of both glial markers was significantly increased compared to that observed in sham animals (p < 0.01). Regarding GFAP expression, GSE treatment progressively reduced the expression of this marker as the dose tested increased, with the 20 mg/kg dose of GSE showing significant differences in this marker compared to saline-treated SCI animals (p < 0.05) (Fig. 6). In parallel, both doses of CE completely prevented GFAP overexpression in SCI mice, showing no significant differences from the Sham group (p’s > 0.05). IBA1 expression was also significantly reduced in SCI animals treated with GSE and CE compared to animals treated with saline at all tested doses (all p’s < 0.05). On the one hand, GSE10 and GSE20 showed a reduction of this marker to levels similar to those observed in the sham group (p's > 0.05), whereas the remaining tested doses of both treatments reduced the expression of this marker, although it was still significantly higher than that observed in the sham group (Fig. 6).

### Treatments with polyphenolic extracts attenuate the expression of chemokines and their receptors as well as phosphorylated ERK in the spinal cord of animals with spinal cord contusion

**Figure 7:**
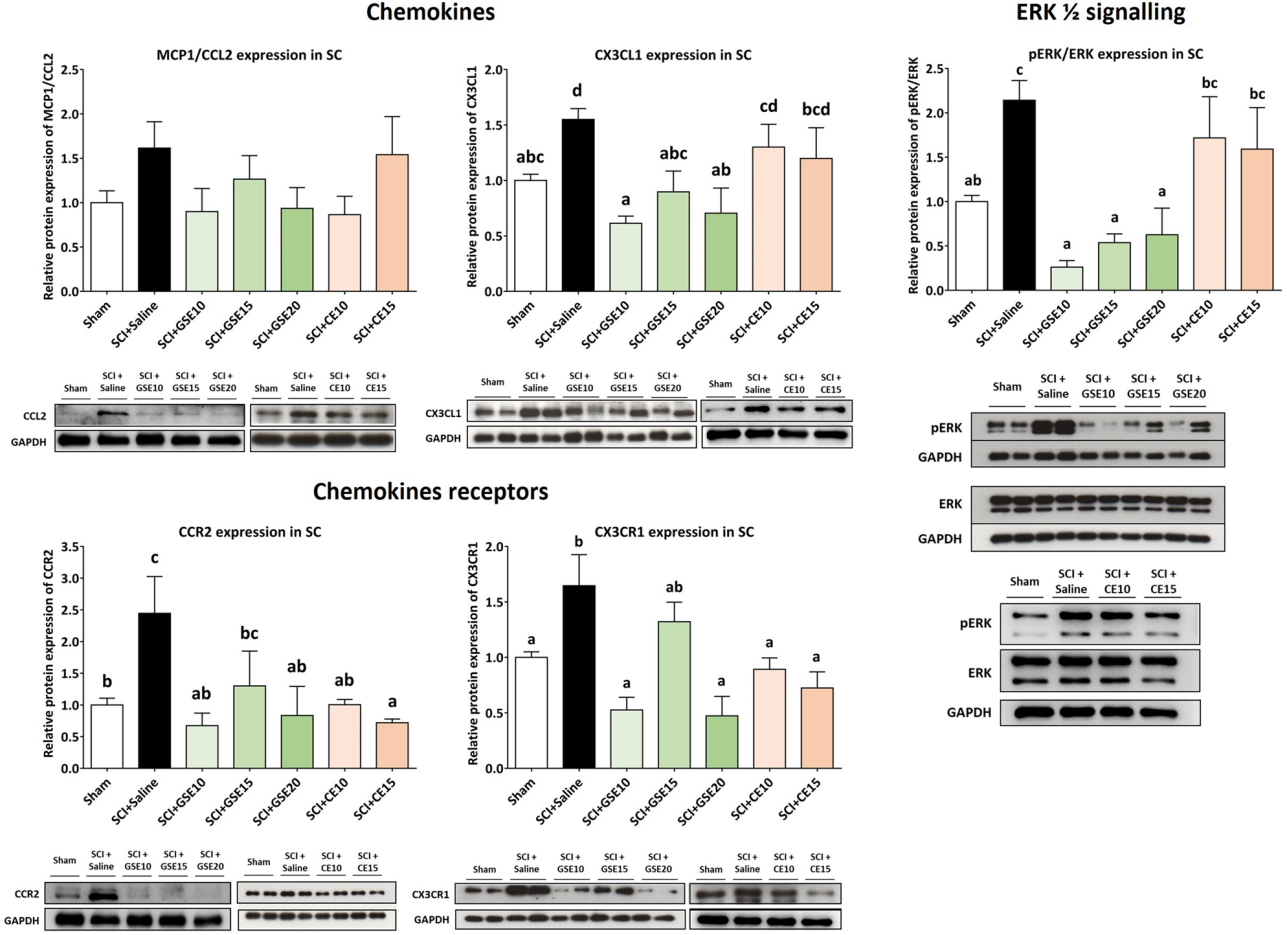
Spinal expression of chemokines (MCP1/CCL2 and CX3CL1) and their receptors (CCR2 and CX3CR1) and spinal levels of ERK phosphorylation after preventive GSE and CE treatment in spinal cord-injured mice at the end of the experimental period. Protein expression was normalized to GAPDH. Data are expressed as a relative percentage with respect to the sham group (mean ± SEM). a–d: Groups not sharing a letter showed significant differences, p < 0.05. Experimental groups: Sham (MCP1/CCL2 n = 9; CCR2 n = 10; CX3CL1 n = 9; CX3CR1 n = 8; pERK/ERK = 9), SCI + Saline (MCP1/CCL2 n = 9; CCR2 n = 9; CX3CL1 n = 8; CX3CR1 n = 7; pERK/ERK = 8), SCI + GSE10 (MCP1/CCL2 n = 5; CCR2 n = 5; CX3CL1 n = 4; CX3CR1 n = 4; pERK/ERK = 4), SCI + GSE15 (MCP1/CCL2 n = 5; CCR2 n = 5; CX3CL1 n = 4; CX3CR1 n = 4; pERK/ERK = 4), SCI + GSE20 (MCP1/CCL2 n = 5; CCR2 n = 5; CX3CL1 n = 4; CX3CR1 n = 4; pERK/ERK = 4), SCI + CE10 (MCP1/CCL2 n = 4; CCR2 n = 5; CX3CL1 n = 5; CX3CR1 n = 5; pERK/ Control images were reused either for illustrative purposes or methodological purposes when several protein levels were assessed in one blot. Full-length blots are presented in Supplementary Figs. S3, S4 and S5.

Although the results showed increased expression of CCL2/MCP1 in the spinal cord of SCI animals at 21 dpi, it was not significant (p > 0.05) compared to sham animals. In contrast, its receptor (CCR2) was significantly overexpressed in the saline group compared to the sham group (p < 0.01). While all tested doses of GSE and CE decreased CCR2 expression when compared to Sham levels (p’s > 0.05), GSE15 did not differ from either Sham or Saline groups (p’s > 0.05), showing a lower efficacy in reducing this chemokine receptor expression in the spinal cord (Fig. 7). In parallel, SCI caused overexpression of CX3CL1 and its receptor CX3CR1 in the spinal cord of saline-treated SCI animals compared to control animals (all p’s < 0.05). While all doses of GSE decreased CX3CL1 expression at sham levels (p's > 0.05), no dose of CE significantly reduced CX3CL1 expression compared to saline-treated SCI animals (p's > 0.05). Similar to the CCR2 results, all doses of GSE and CE decreased CX3CR1 expression up to Sham levels (p’s > 0.05), except for the dose of 15 mg/kg of GSE which did not differ from either Sham or Saline groups (p’s > 0.05), evidencing its lower efficacy in reducing this receptor expression in the spinal cord (Fig. 7). Taken together, these results suggest that GSE treatment modulates the expression of the chemokine CX3CL1 and the chemokine receptors CX3CR1 and CCR2 in the injured spinal cord, whereas CE treatment modulates the expression of these receptors.

Moreover, SCI animals treated with saline solution showed a significant increase in the expression of phosphorylated ERK compared to the control animals (p < 0.05). Treatment with GSE significantly reduced this expression to levels equal to those observed in the sham group (p’s > 0.05). This pattern was not observed in CE-treated animals since none of the two tested doses reduced ERK phosphorylation compared to SCI animals treated with saline solution (p’s > 0.05) (Fig. 7).

### Treatments with polyphenolic extracts tend to modulate gliosis and CX3CL1/CX3CR1 expression in the anterior cingulate cortex (ACC) and periaqueductal gray (PAG) areas of mice subjected to spinal cord contusion

**Figure 8:**
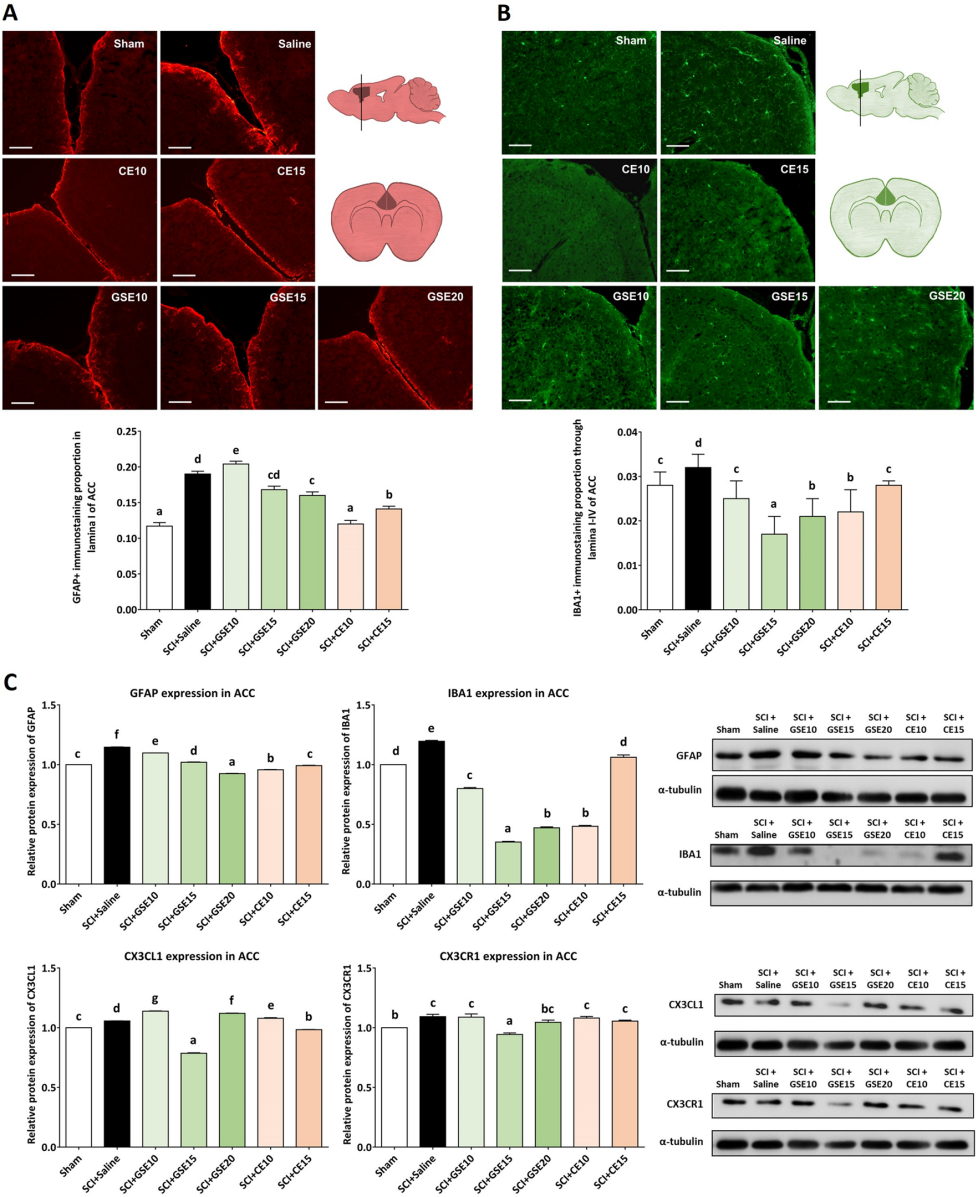
Effects of GSE and CE treatment on gliosis and CX3CL1/CX3R1 expression in the ACC of SCI mice at the end of the experimental period. (**A**) Representative histological images of the ACC immunostained against GFAP of each group (scale bar 200 μm) and histogram representing the proportion of GFAP immunoreactivity in ACC. Data are expressed as the mean ± SEM. (**B**) Representative histological images of the ACC immunostained against IBA1 of each group (scale bar 200 μm) and histogram representing the proportion of IBA1 immunoreactivity in ACC. Data are expressed as the mean ± SEM. (**C**) GFAP, IBA1, CX3CL1 and CX3CR1 expression in ACC. Protein expression was normalized to a-tubulin. Data are expressed as a relative percentage with respect to the sham group (mean ± SEM). Control images were reused either for illustrative purposes or methodological purposes when several protein levels were assessed in one blot. Full-length blots are presented in Supplementary Fig. S6. a–g: Groups not sharing a letter showed significant differences, p < 0.05. Experimental groups: Sham (GFAP n = 5; IBA1 n = 5; CX3CL1 n = 4; CX3CR1 n = 4), SCI + saline (GFAP n = 5; IBA1 n = 5; CX3CL1 n = 4; CX3CR1 n = 4), SCI + GSE10 (GFAP n = 5; IBA1 n = 5; CX3CL1 n = 4; CX3CR1 n = 4), SCI + GSE15 (GFAP n = 5; IBA1 n = 5; CX3CL1 n = 4; CX3CR1 n = 4), SCI + GSE20 (GFAP n = 5; IBA1 n = 5; CX3CL1 n = 4; CX3CR1 n = 4), SCI + CE10 (GFAP n = 5; IBA1 n = 5; CX3CL1 n = 4; CX3CR1 n = 4), SCI + CE15 (GFAP n = 5; IBA1 n = 5; CX3CL1 n = 4; CX3CR1 n = 4).

In histological sections of the anterior cingulate cortex (ACC), GFAP immunostaining was observed in the neural parenchyma but also in the edges forming the glia limitans (Fig. 8A), whereas IBA1 immunostaining for reactive and nonreactive microglial cells was seen in the parenchyma of the ACC (Fig. 8B). The immunohistochemical results indicated a significant increase in both GFAP (p < 0.001) and IBA1 (p < 0.05) immunoreactivity proportions in the ACC of SCI animals treated with saline solution when compared with sham, at 21 dpo. Both CE10 and CE15 and only GSE20 showed a significant reduction in the GFAP immunoreactivity proportion in the ACC compared to the saline group. In contrast, all doses of GSE and CE treatments significantly reduced microgliosis in the ACC compared to the group treated with saline solution (p < 0.05) up to levels similar or lower than those observed in the sham group (Fig. 8B). For molecular assessments, western blot analysis confirmed a significant upregulation of GFAP and IBA1 expression in the ACC of mice subjected to SCI and treated with saline solution. Treatment with either GSE or CE significantly reduced the expression of GFAP and IBA1 in the ACC compared to SCI animals treated with saline solution (p < 0.05), up to levels equal to or lower than those observed in the control group Sham (Fig. 8C). Finally, CX3CL1 and its receptor, CX3CR1, were significantly overexpressed in the ACC of the saline group compared with the sham group (p < 0.05). GSE15 showed a significant reduction in the expression of both CX3CL1 and CX3CR1 in ACC compared with the saline group (p’s < 0.05). Regarding CE treatment, only CE15 showed a reduction in CX3CL1 expression below sham levels (p < 0.05) (Fig. 8C).

**Figure 9:**
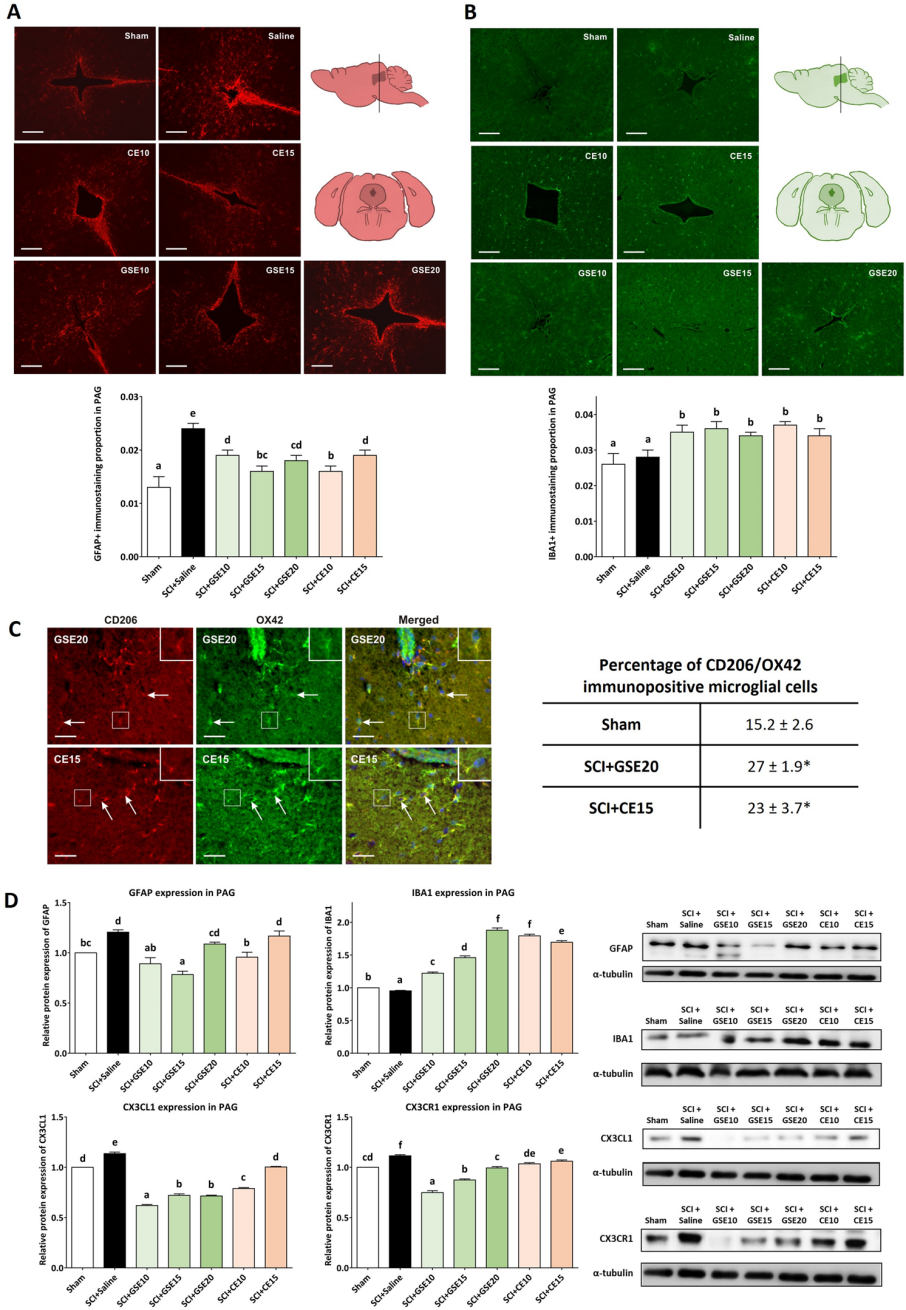
Effects of GSE and CE treatment on gliosis and CX3CL1/CX3R1 expression in the PAG of SCI mice at the end of the experimental period**.** (**A**) Representative histological images of the PAG immunostained against GFAP of each group (scale bar 200 μm) and histogram representing the proportion of GFAP immunoreactivity in PAG. Data are expressed as the mean ± SEM. (**B**) Representative histological images of the PAG immunostained against IBA1 of each group (scale bar 200 μm) and histogram representing the proportion of IBA1 immunoreactivity in PAG. Data are expressed as the mean ± SEM. (**C**) Percentage of CD206 immunopositive OX42 immunostained microglial cells in PAG of animals with SCI and treated with GSE (20 mg/kg) and CE (15 mg/kg) compared with sham-operated control. * p < 0.01 compared with Sham. Representative pictures illustrating double immunostaining of OX42-immunopositive microglial cells (**A**) and CD206 (**B**) in the PAG sections of mice subjected to SCI and treated with GSE20. Merged green (OX42-FITC) and red (CD206-TRITC) immunofluorescence and blue Hoechst nuclear staining indicate the position of OX42 + /CD206 + microglial cells (**C**). Scale bar = 50 µm. (**D**) GFAP, IBA1, CX3CL1 and CX3CR1 expression in PAG. Protein expression was normalized to a-tubulin. Data are expressed as a relative percentage with respect to the sham group (mean ± SEM). Control images were reused either for illustrative purposes or methodological purposes when several protein levels were assessed in one blot. Full-length blots are presented in Supplementary Fig. S7. a–g: Groups not sharing a letter showed significant differences, p < 0.05. Experimental groups: Sham (GFAP n = 5; IBA1 n = 5; CX3CL1 n = 6; CX3CR1 n = 6), SCI + saline (GFAP n = 5; IBA1 n = 5; CX3CL1 n = 6; CX3CR1 n = 6), SCI + GSE10 (GFAP n = 5; IBA1 n = 5; CX3CL1 n = 6; CX3CR1 n = 6), SCI + GSE15 (GFAP n = 5; IBA1 n = 5; CX3CL1 n = 6; CX3CR1 n = 6), SCI + GSE20 (GFAP n = 5; IBA1 n = 5; CX3CL1 n = 6; CX3CR1 n = 6), SCI + CE10 (GFAP n = 5; IBA1 n = 5; CX3CL1 n = 6; CX3CR1 n = 6), SCI + CE15 (GFAP n = 5; IBA1 n = 5; CX3CL1 n = 6; CX3CR1 n = 6).

In histological sections of periaqueductal gray (PAG), GFAP- and IBA1-positive cells were also detected for all experimental groups (Fig. 9A). Regarding astrogliosis, the saline group showed a significant increase in the proportion of GFAP immunoreactivity compared to the sham group (p < 0.001), indicating that SCI induced astroglial activation in the PAG at 21 dpi. For GSE and CE treatments, all tested doses reduced GFAP reactivity in PAG when compared with Saline group (all p’s < 0.01) although none of them reached at immunoreactivity Sham levels (all p’s < 0.05). CE10 and GSE15 showed the lowest astroglial reactivity in PAG, followed by GSE20, GSE10 and CE15. In contrast to the spinal cord and ACC, the proportion of IBA1 immunoreactivity in the PAG did not differ between the sham and saline groups (p > 0.05), indicating that SCI in mice did not induce microgliosis in the PAG. Furthermore, none of the GSE and CE treatments reduced this immunolabeling, but in addition, the SCI groups treated with GSE or CE showed a significant increase in IBA1 immunoreactivity compared to the Sham and Saline groups (all p's < 0.05) (Fig. 9B). For molecular assessments, western blot analysis showed a significant up-regulation of GFAP expression and down-regulation of IBA1 expression in PAG of mice subjected to SCI and treated with saline solution when compared with Sham (p’s < 0.01). While GSE10, GSE15 and CE10 treatments significantly showed a GFAP expression reduction in PAG compared with saline-treated SCI animals (p < 0.05), GSE20 and CE15 did not. In contrast, the GSE and CE treatment groups showed IBA1 upregulation compared to the sham group (all p’s > 0.05) (Fig. 9D). This increase in microglial markers was probably due to an increased M2 subtype of microglial cells, as demonstrated by double immunostaining with CD206 (Fig. 9C). Finally, the expression of CX3CL1 and its receptor (CX3CR1) also increased in the PAG of mice subjected to SCI and treated with saline solution in comparison with control mice (sham group) (p’s < 0.05), and treatments with GSE and CE decreased the expression of both markers (p’s < 0.05) (Fig. 9D).

Altogether, these results suggest that SCI causes gliosis in supraspinal structures involved in pain processing, such as the ACC and PAG, and that treatment with either GSE or CE modulates this glial reactivity and reduces the expression of CX3CL1 and CX3CR1 in the ACC and PAG, except for CE treatment in the ACC, which has no effect on the expression of these two markers.

## Discussion

The present manuscript reports that both GSE and CE treatments administered during the acute phase of spinal cord injury exert preventive effects on both thermal hyperalgesia and mechanical allodynia development in mice subjected to spinal cord contusion. Both extracts also reduce gliosis in the spinal cord and in supraspinal areas, such as the anterior cingulate cortex (ACC) and periaqueductal gray matter (PAG), two areas implicated in pain processing. Moreover, GSE and CE treatments also decreased CX3CL1, CCR2 and CX3CR1 expression in the spinal cord of SCI animals and CX3CL1 and CX3CR1 expression in ACC and PAG supraspinal areas. All these findings suggest that GSE and CE alleviate neuropathic pain development after SCI by modulating the reactivity of glial cells and the levels of chemokines, as well as the expression of their receptors, at both the spinal and supraspinal levels. It is worth mentioning also that our findings indicated that the ventrolateral funiculus was preserved on both sides of spinal cord. Traumatic spinal cord injuries lead to histological heterogeneity of the spinal cord parenchyma injury associated with variable pain responses, but when the ventrolateral funiculus is preserved after spinal cord injury, neuropathic pain may develop^35–37^. Considering that after mild SCI the ventrolateral funiculus was preserved in all groups, it can be suggested that antinociceptive effects observed would be associated with the GSE and CE treatments and not because of the severity of the injury effecting this region that it is necessary to preserve nociception responses. Overall, to our knowledge, this is the first study to use GSE and CE in an experimental model of spinal cord contusion-induced central neuropathic pain.

HPLC analysis showed that the major polyphenols present in the obtained GSE were gallic acid, protocatechuic acid and catechin, whereas chlorogenic acid, neochlorogenic acid, and cryptochlorogenic acid were the major polyphenols in the CE. Given the data obtained, it is not unreasonable to consider that the antinociceptive effects of the plant extracts used in the present study may be attributed to the antinociceptive effects of the polyphenols contained in them. Considering that obtaining the high temperature during the extract denatures the potential enzymes or proteins present in the extract and that filtration and sterilization with a 0.22 μm filter avoids the presence of bacteria in the final solution, it is reasonable to hypothesize that the polyphenol content in the final solution would be responsible for the effects of the extracts. In this context and in relation to the vegetable extract of the grape stalk, it has been reported that administration of gallic acid, protocatechuic acid or catechin may reduce pain responses in different animal models of peripheral neuropathic pain, such as chronic constriction injury (CCI)^38^, spinal nerve ligation^39^, paclitaxel-induced neuropathic pain^40^, and inflammatory pain^38,39^. Regarding the major polyphenols in the coffee extract, it has been shown that chlorogenic acid treatment alleviates pain responses after CCI^41,42^ and diabetic neuropathy^43^. Moreover, infusion of aerial parts of *Salvia chudaei Batt. & Trab* containing 4-O-caffeoylquinic acid (cryptochlorogenic acid) alleviates painful responses after carrageenan, acetic acid and formalin injection^44^. Altogether, this evidence indicates that the polyphenolic compounds present in both extracts used in this study have antinociceptive effects in different experimental models of pathological pain, although very few of them have been used in an experimental model of central neuropathic pain. However, it is also worth mentioning that some of the major polyphenols contained in both extracts have also been shown to exert neuroprotective effects on the injured spinal cord, including gallic acid^45,46^, protocatechuic acid^47^ and chlorogenic acid^48^.

Regarding the biological mechanisms of polyphenols, they are known to exert changes in the nervous system that may lead to pain relief. In this context, the present study shows that both polyphenol-rich plant extracts exert a reduction in SCI-related gliosis, and considering the well-described relationship between spinal cord astro- and microgliosis with CNP^6^, it can be suggested that these modulatory effects contribute to a reduction in reflexive pain responses. Interestingly, this dose-dependent decrease in gliosis has also been observed in supraspinal structures involved in pain processing in the anterior cingulate cortex (ACC) and periaqueductal gray (PAG) areas. These effects of both extracts on the reactivity of microglial cells and astrocytes after spinal cord contusion may be attributed to the polyphenolic compounds contained in the extract according to available data^8^, although no previous studies specifically showed SCI-related gliosis modulation after the administration of these major polyphenols. However, other studies have shown that these polyphenols exert similar effects on glial cells, such as those reporting that gallic acid^49^, protocatechuic acid^50,51^, chlorogenic acid^52^, and neochlorogenic acid^53^ reduce the reactivation of BV2 microglial cells stimulated with LPS. In addition, other polyphenols have been shown to exert gliosis modulation in the central nervous system of animal models of Parkinson-like disease^54^, cerebral ischemia^55^, neuropathy^56^ and Alzheimer-like disease^57^. On the other hand, a significant increase in microgliosis was observed after GSE and CE treatments only in PAG, in this case due to an increase in the M2 subtype (Fig. 9C), and therefore adopting cellular contents with anti-inflammatory functions. These results are consistent with the published regional heterogeneity of microglial activation and modulation in the central nervous system^58,59^.

Regarding activated glial mechanisms, it is known that these cells release inflammatory mediators that induce hyperexcitability of spinal nociceptive neurons, enhance the release of neurotransmitters from nociceptive afferent fibers that project into the dorsal horn of the spinal cord, and facilitate central sensitization of spinal nociceptive neurons^60^. These are all cellular phenomena that potentiate the transmission of action potentials through the nociceptive somatosensory system, generating responses of thermal hyperalgesia and mechanical allodynia, the two main symptoms of neuropathic pain^61^. Consequently, the reduction of gliosis by the polyphenolic extracts tested in the present study may contribute to reducing the expression and release of these inflammatory mediators that induce neuropathic pain development after SCI. In this context, to elucidate associated mechanistic insights, the expression of chemokines (CCL2 and CX3CL1) and their receptors (CCR2 and CX3CR1) associated with neuropathic pain development have been studied, and a significant modulation of them after extract treatment was observed. For CCR2, our results show its upregulation in non-treated SCI animals, a result supported by previous studies showing CCR2 overexpression in spinal cord injured rats^36^. CCR2 is expressed on astrocytes, microglia, and neurons^62,63^, and it is known that the chemokine CCL2 acts directly on CCR2-positive excitatory neurons to regulate central sensitization via postsynaptic NMDARs^64^, facilitating spinal synaptic transmission. Hence, although no significant changes have been observed in CCL2 expression, the significant reduction in CCR2 may attenuate spinal hypersensitivity development. Regarding the fractalkine pathways, the present work also demonstrates that mild SCI in mice causes overexpression of both CX3CL1 and CX3CR1 in the spinal cord at 21 dpi, as well as their significant reduction after either GSE or CE treatment. It has been previously reported that CX3CR1 is upregulated in microglia, astrocytes, and neurons^65^, and it is known that intrathecal administration of CX3CL1 produces dose-dependent mechanical allodynia and thermal hyperalgesia^66^. It is known also that soluble fractalkine (sFKN) induces nociceptive behaviors after activation of the CX3CR1 receptor on microglia, activation of p38 mitogen-activated protein kinase (MAPK)-mediated pathways^67^ and release of inflammatory mediators (e.g., IL-1beta, IL6, nitric oxide)^68^, which contribute to central sensitization. The plasma membrane of neurons in the dorsal horn of the spinal cord contains fractalkine, which is cleaved from neuronal membranes by the action of cathepsin S (CatS) released by reactive microglial cells, forming soluble fractalkine (sFKN)^69^, which in turn stimulates microglial cells by inducing the synthesis and release of pain-inducing inflammatory messengers, as described above. In summary, all these findings suggest that both the CCL2/CCR2 and CX3CL1/CX3CR1 pathways are implicated in the generation of neuropathic pain^70^, and consequently, their modulation by GSE and CE may contribute to the alleviation of reflexive pain response development after SCI.

In addition to chemokines expression modulation, our results also showed a significant modulation of pERK1/2 overexpression in the spinal cord in GSE animals, which was overexpressed in SCI animals. ERK phosphorylation has been previously reported in dorsal horn nociceptive neurons and in reactive astrocytes^22^ accompanied by increased pain hypersensitivity after spinal cord injury^71^. Thus, since GSE tested doses completely prevented the SCI-related upregulation of pERK, it can be suggested that polyphenols in this extract may modulate the ERK signaling pathway, contributing to neuropathic pain development attenuation. Indeed, several phenolic compounds have been shown to modulate the MAPK pathway by acting at various steps of the activation cascade and consequently on downstream effectors^72^. Specifically, several ex vivo studies have shown the ability of gallic acid, a GSE polyphenol in GSE, to inhibit ERK phosphorylation in human ovarian carcinoma cells^73^, retinal capillary endothelial cells^74^ and breast cancer cells^75^.

In addition to the results described in the spinal cord, changes after SCI in supraspinal structures involved in pain processing have been described in the present work. Concretely, there was an increase in gliosis and levels of CX3CL1/CX3CR1 in both ACC and PAG and a reduction in gliosis and expression of this chemokine and its receptor after treatment with polyphenolic extracts. Previously, changes in CCL2 and CCL3 expression have been described in the ACC and PAG after spinal cord contusion^76^, but no previous studies have described gliosis and changes in the expression of CX3CL1/CX3CR1 in the ACC and PAG after SCI. Fractalkine and its receptor form a crosstalk axis between neurons and microglial cells in the development and maintenance of neuropathic pain, leading to the release of inflammatory mediators from microglial cells (e.g., cytokines, prostaglandins, nitric oxide) that contribute to hyperexcitability of nociceptive neurons at the postsynaptic level, enhance the release of other pro-nociceptive neurotransmitters at the presynaptic level, and facilitate central sensitization of nociceptive neurons^77–79^. The neurons of ascending nociceptive pathways that have become hyperexcited at the level of the spinal cord as a result of the injury and the consequent depolarization or hyperdepolarization of the series of neurons involved in propagation of nociceptive information towards supraspinal structures may contribute to the activation of microglial cells in these supraspinal pain processing structures. The nociceptive neurons of the spinothalamic tract possess glutamate as a neurotransmitter, which they release onto the third-order neurons of the supraspinal pain-processing structures^80^. The nociceptive neurons of the thalamic nuclei that project to the anterior cingulate cortex also release glutamate^81^. Microglial cells express glutamatergic receptors^82^, which when activated by glutamate promote an increase in extracellular calcium influx^83^. In addition, activation of NMDA receptors and group III metabotropic glutamate receptors induces intracellular Ca^2+^ release from the endoplasmic reticulum in microglial cells^84^, and it is well known that CatS released by microglial cells requires both extracellular calcium influx and mobilization of intracellular calcium^77^. All these findings suggest that glutamate released by nociceptive neurons may also stimulate PAG and ACC microglial cells, causing the synthesis and release of CatS, a protease that will induce neuronal membrane fractalkine breakdown, generating soluble fractalkine, which, in turn, acts again on the fractalkine receptors of microglial cells, facilitating the synthesis and release of inflammatory mediators that favor neuropathic pain in these supraspinal structures. Hence, considering that polyphenols have been reported to modulate metabotropic glutamate activity in preclinical models^85,86^, it can be suggested that polyphenols contained in either GSE or CE may modulate glutamatergic synapses, leading to central sensitization process modulation. Concretely, since the ascending nerve pathway from the spinal cord to the supraspinal structures is a glutamatergic pathway, it would be expected that some of these polyphenols could modulate this glutamatergic neurotransmission of the ascending nociceptive pathway and thus the transmission of action potentials and the reactivation of glial cells.

As to potential translation of these findings into the clinics, it is worth mentioning that all these beneficial effects exerted by GSE and CE are exerted without systemic toxicity since no weight-loss neither hepatotoxic or nephrotoxic side effects were observed. Alanine aminotransferase (ALT) and aspartate aminotransferase (AST) have been widely used as sensitive markers of possible liver toxicity in non-clinical toxicology studies and clinical trials, Transaminase activity in the blood (serum or plasma) is increased especially in hepatocellular damage induced by drugs or diseases^87,88^. On the other hand, blood accumulation of urea/bun is a sign of nephrotoxicity since the kidneys tend to purify these compounds. Several traditional tests of renal dysfunction and damage, including plasma creatinine and urea, are used in the first instance to detect nephrotoxicity^89,90^. Thus, the lack of significant increase of these biomarkers after GSE or CE administration suggest that such treatments may be safety.

Finally, although all these findings may be considered promising, we are aware of some limitations that may be addressed in future experiments. For instance, it can be shown in the results that the administration of highest doses of GSE and CE were not always showing the best results on CX3CL1 or astrogliosis modulation on ACC. Although it could be speculated the potential role of neuroprotective effects of CX3CL1/CX3CR1 pathways^91,92^, it could be also explained by the fact that high doses exceed the selectivity threshold of polyphenols for their primary target. However, considering that primary targets of polyphenols have been not yet elucidated, further experiments are needed for this purpose. On the other hand, this study was focused on female sex since it is known that females show higher prevalence of chronic pain and higher vulnerability in the development of comorbid pain and emotional disorders^93,94^. However, considering evidence suggesting that the analgesic effects of plant extracts or solutions of polyphenols could be influenced by gender^95,96^, the effects of GSE and CE may be also studied in male in future experiments.

## Conclusions

In conclusion, our findings indicate that preventive treatment by repeated administration of either GSE or CE after injury attenuates CNP development in wild-type CD-1 Swiss female mice during the acute phase of SCI without hepatotoxic or nephrotoxic effects. GSE and CE pharmacological effects may be associated with gliosis modulation in both spinal cord and supraspinal structures involved in pain processing, as well as the prevention of upregulation of pathological pain development-related chemokines and their receptors. As mentioned before, considering that the high temperature used during the extract obtaining may denature protein molecules and considering that 0.22 μm filtration avoids the presence of bacteria in the final solution, it is reasonable to hypothesize that polyphenol content in the final solution would be responsible for the effects of the extracts.

Based on these results, the repeated administration of either grape stalk extract or decaffeinated coffee extract following SCI are suggested to be potential pharmacological strategies to prevent spinal cord injury-induced pathological pain development. These strategies can extend the possibilities described so far, based on natural products (see Supplementary Table S1).

## Supplementary Information

Supplementary Information.

## Data Availability

All data generated or analyzed during this study are included in this published article and its supplementary information files.

## Abbreviations

ACC: Anterior cingulate cortex
ALT/GPT: Alanine aminotransferase
AST/GOT: Aspartate aminotransferase
BMS: Basso Mouse Scale
BUN: Blood urea nitrogen
CE: Coffee extract
CNP: Central neuropathic pain
GAPDH: Glyceraldehyde-3-phosphate dehydrogenase
GFAP: Glial fibrillary acidic protein
GSE: Grape stalk extract
HPLC: High-performance liquid chromatography
IBA1: Ionized calcium-binding adaptor molecule 1 IgG: immunoglobulin
PAG: Periaqueductal gray matter
PBS: Phosphate-buffered saline
SCI: Spinal cord injury
SEM: Standard error of the mean
