# Supplementary material for: Polyphenolic grape stalk and coffee extracts attenuate spinal cord injury-induced neuropathic pain development in ICR-CD1 female mice
Source: Sci Rep. 2022 Sep 2;12:14980. doi: 10.1038/s41598-022-19109-4 (PMC9440260; doi:10.1038/s41598-022-19109-4)
Supplement: Supplementary file 1 — Supplementary Information. [file 41598_2022_19109_MOESM1_ESM.pdf]

Supplementary Material

Polyphenolic grape stalk and coffee extracts attenuate spinal cord injury-induced neuropathic pain development in ICR-CD1 female mice

Anna Bagó-Mas[1], Andrea Korimova[2], Meritxell Deulofeu[1], Enrique Verdú[1], Núria Fiol[3], Viktorie Svobodová[2], Petr Dubový[2*], Pere Boadas-Vaello[1*]

[1] Research Group of Clinical Anatomy, Embryology and Neuroscience (NEOMA), Department of Medical Sciences, University of Girona, Girona, Spain.

[2] Department of Anatomy, Division of Neuroanatomy, Faculty of Medicine, Masaryk University, Brno, Czechia.

[3] Department of Chemical Engineering, Agriculture and Food Technology, Polytechnic School, University of Girona, Girona, Spain.

*** Corresponding authors** Dr. Pere Boadas-Vaello and Petr Dubový. Address for correspondence pere.boadas@udg.edu and pdubovy@med.muni.cz

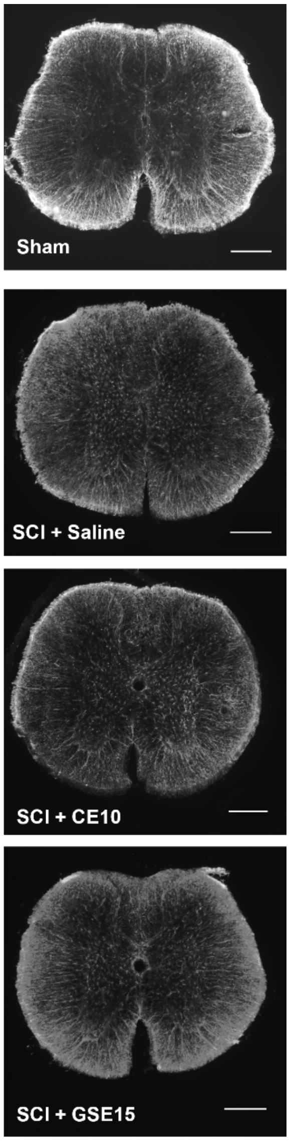

Cross-sections of spinal cord from different experimental groups immunolabeled for GFAP. From top to bottom, cross sections of the spinal cord of animals from the Sham, SCI+Saline, SCI+CE10 and SCI+GSE15 groups are observed. In all these sections, the preservation of the medullar tissue parenchyma can be observed. It should be noted that in all of them the ventrolateral funiculus is preserved on both sides, a spinal cord region necessary to preserve nociception. Scale bar = 250 µm.

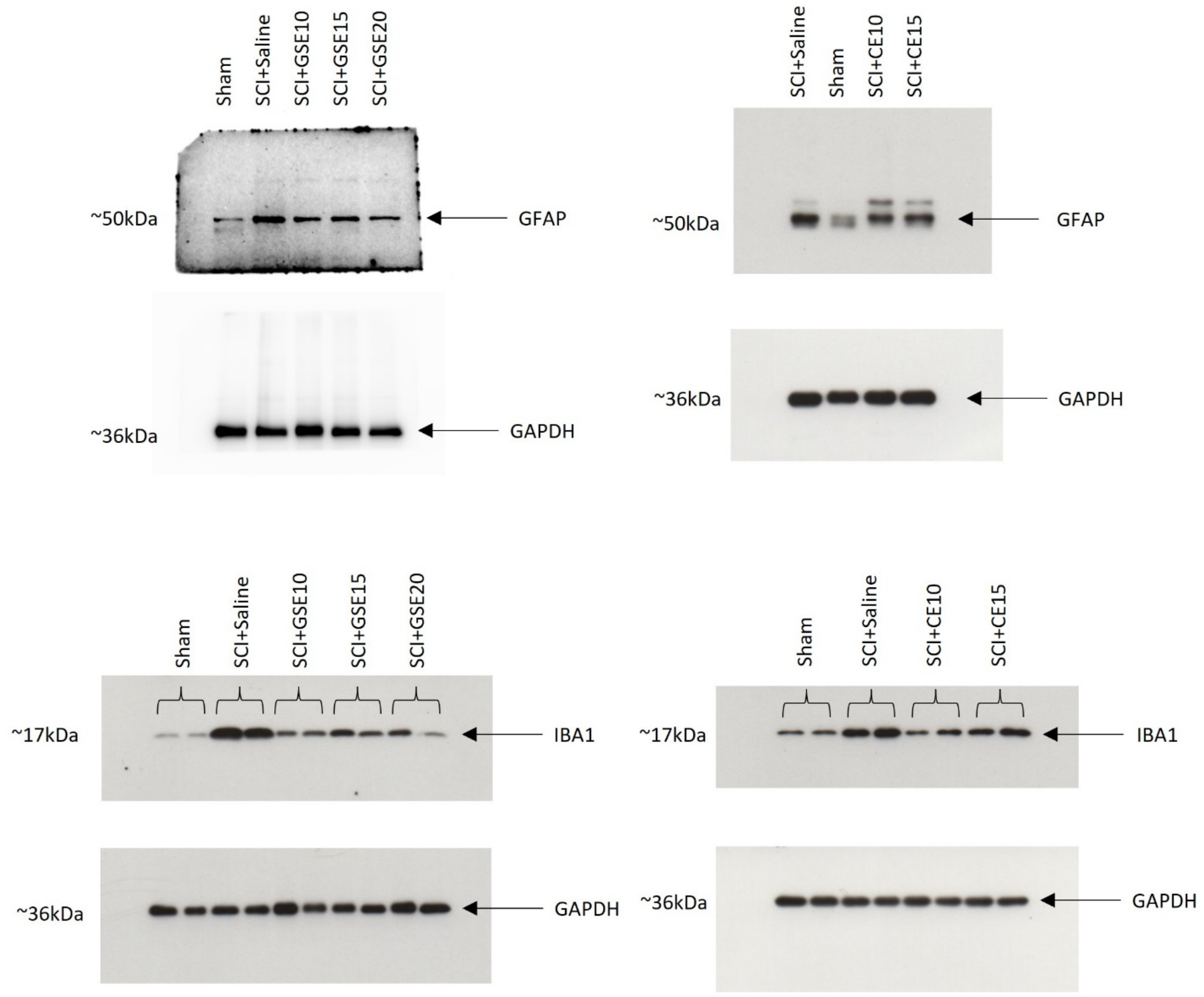

Supplementary Figure S2. Original scanned/revealed full blots for GFAP, IBA1 and respective GAPDH expression in spinal cord shown in Figure 6.

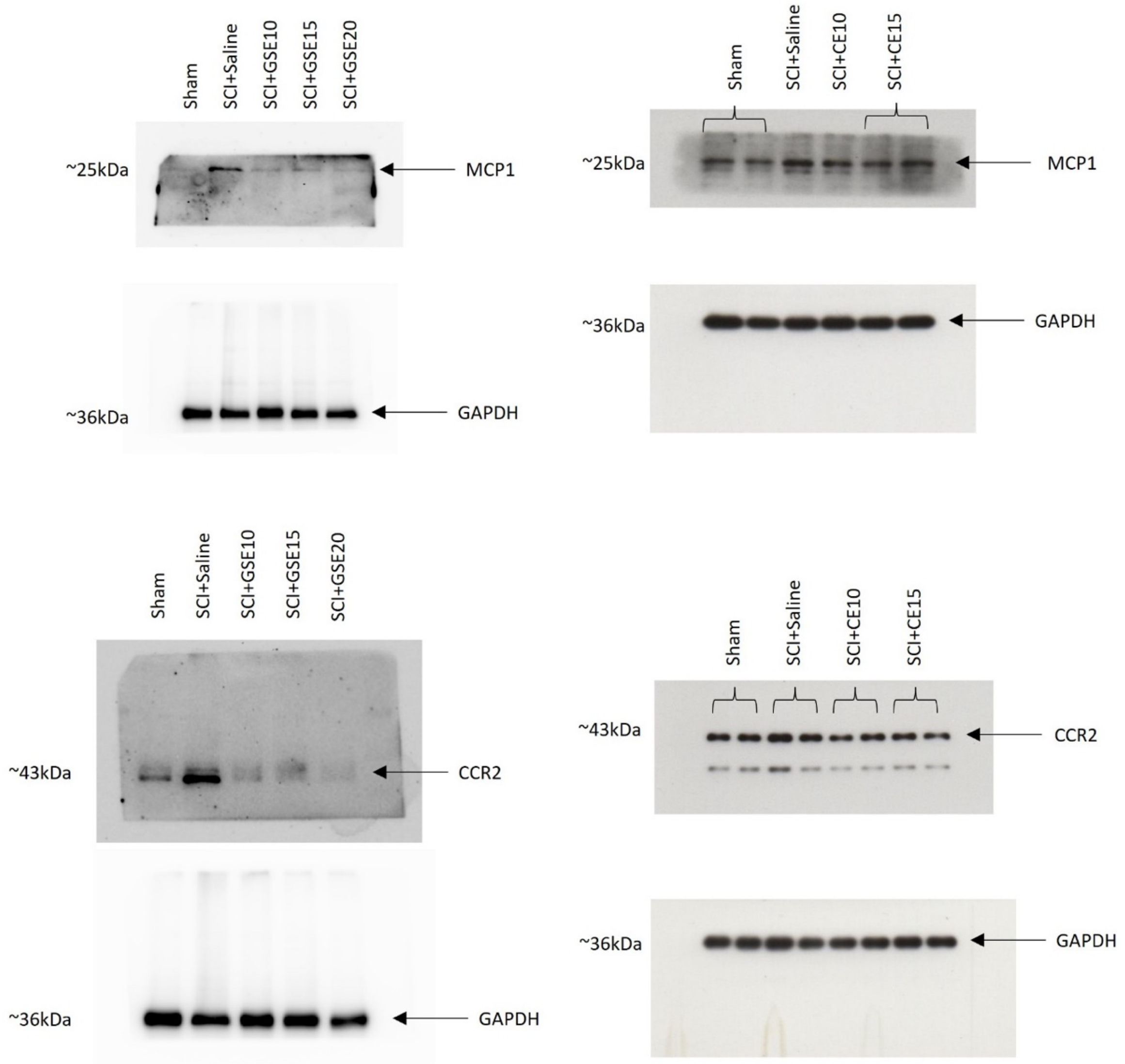

Supplementary Figure S3. Original scanned/revealed full blots for MCP1, CCR2 and respective GAPDH expression in spinal cord shown in Figure 7. Note that developed film blot of spinal cord samples from CE treatment for MCP1 antibody was cut to obtain the image that appears in the Figure 7 of the manuscript, to have an image with a single sample for Sham, SCI+Saline, SCI+CE10 and SCI+SCE15.

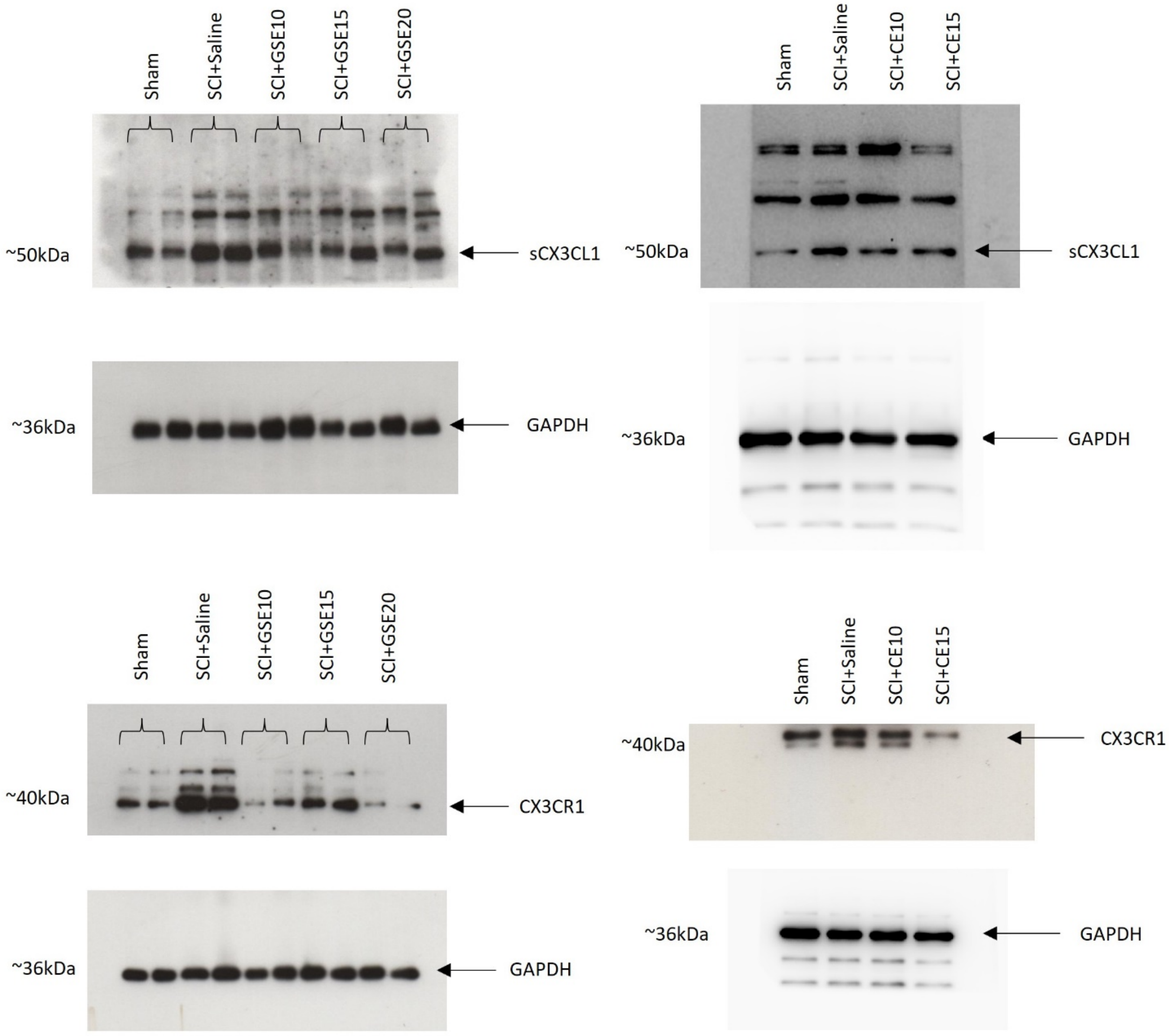

Supplementary Figure S4. Original scanned/revealed full blots for CX3CL1, CX3CR1 and respective GAPDH expression in spinal cord shown in Figure 7.

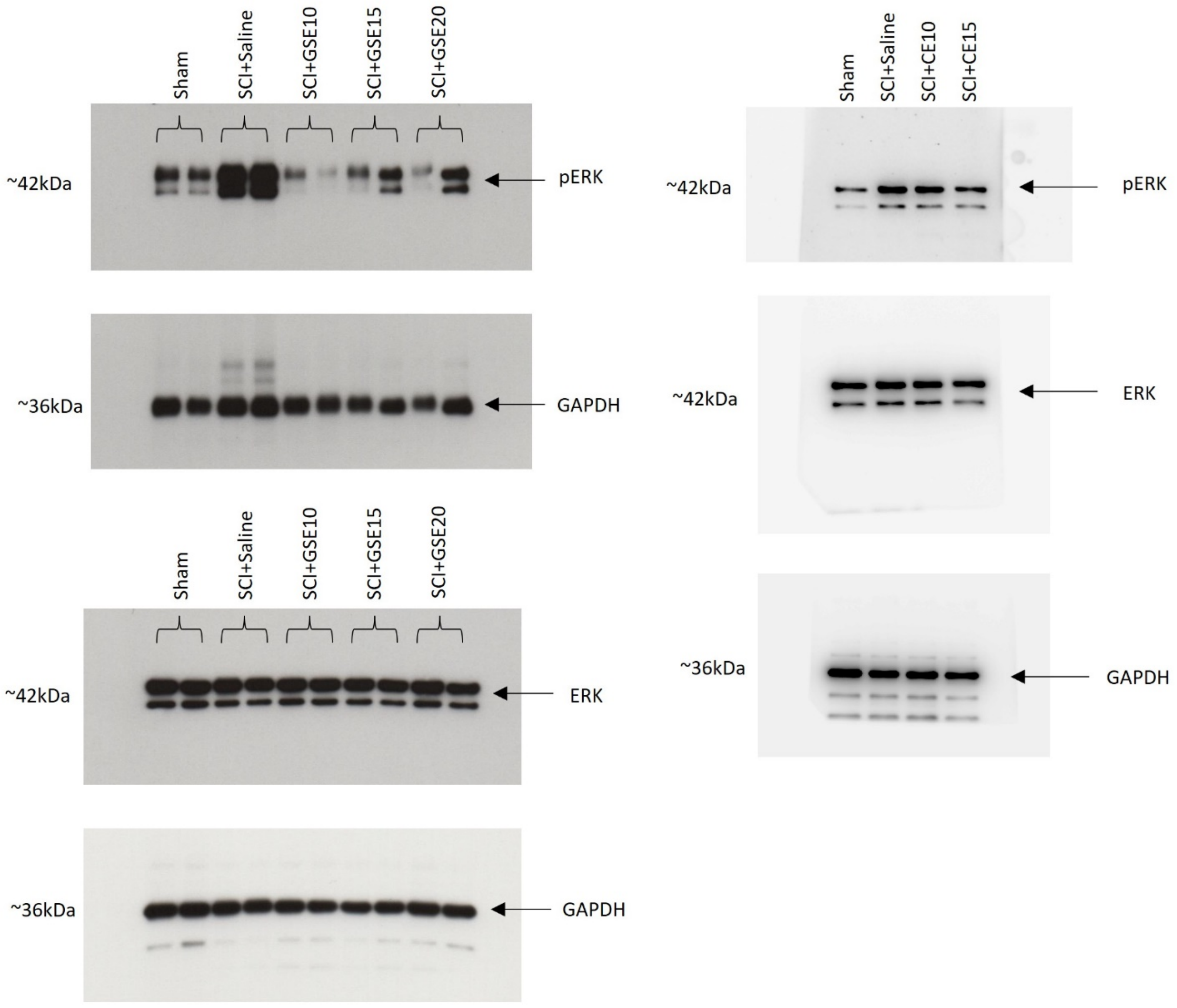

Supplementary Figure S5. Original scanned/revealed full blots for pERK, ERK and respective GAPDH expression in spinal cord shown in Figure 7.

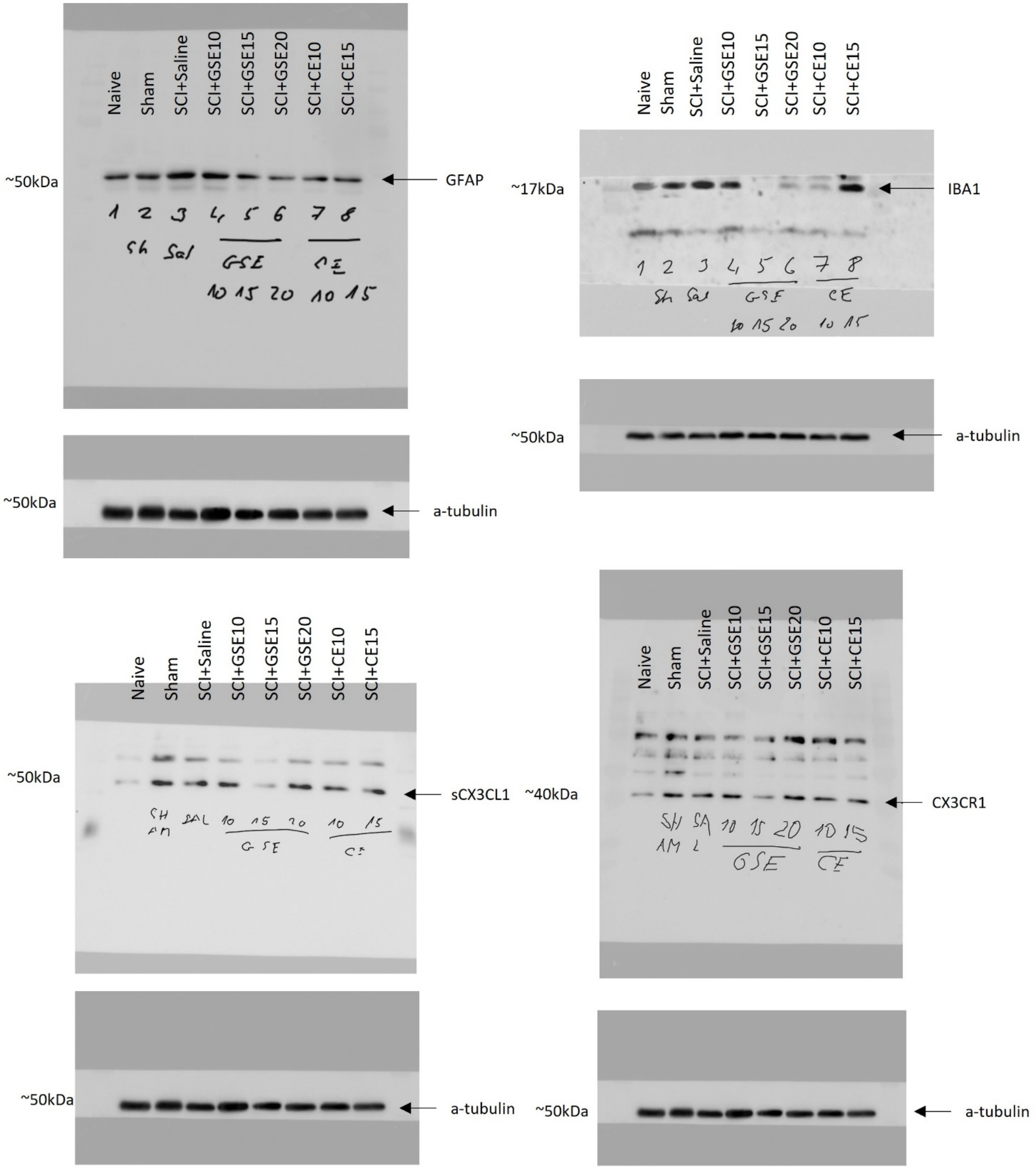

Supplementary Figure S6. Original scanned full blots for GFAP, IBA1, CX3CL1 and CX3CR1 and respective a-tubulin expression in ACC shown in Figure 8.

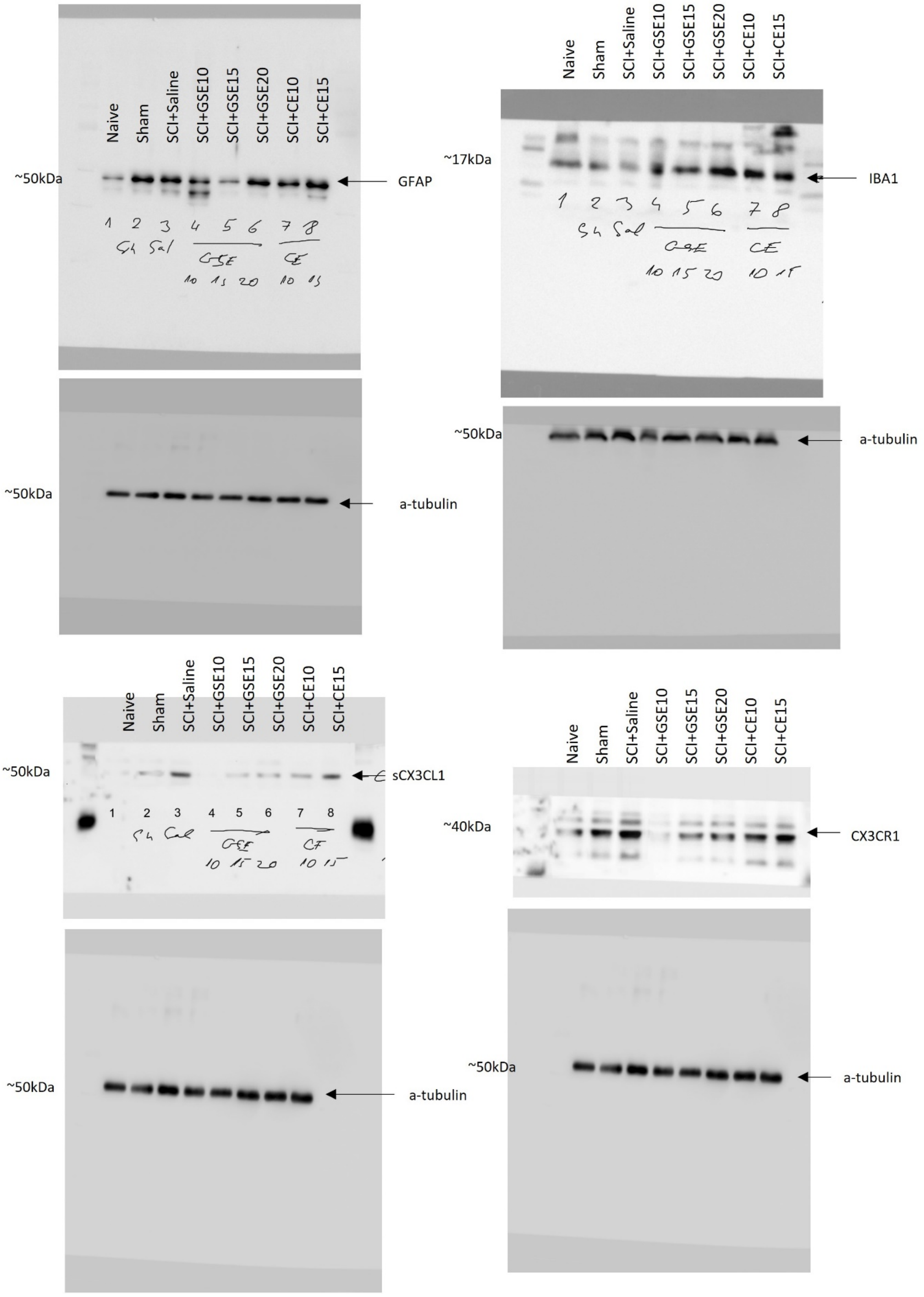

Supplementary Figure S7. Original scanned full blots for GFAP, IBA1, CX3CL1 and CX3CR1 and respective a-tubulin expression in PAG shown in Figure 9.

Supplementary Table S1.

Plant extract or polyphenol treatment for alleviating neuropathic pain after spinal cord injury

Floors	Plant extract	Animal	SCI	Results	Reference
Red ginseng	Total saponin extract (TSE); 50 mg/kg	Male Sprague–Dawley rat	Contusion at T9	Allodynia and hyperalgesia were significantly alleviated by TSE 1h after administration	[1]
Crocus sativus	Crocin; 150 mg/kg	Female Wistar rat	Contusion at T9	Treatment alleviates mechanical allodynia but not thermal hyperalgesia	[2]
Harpagophytum procumbens	Harpagophytum procumbens extract (HPE); 300 mg/kg	Male Sprague–Dawley rat	Contusion at T10	HPE alleviates mechanical allodynia	[3]
Refined from several Chinese herbal medicines, such as Radix Paeoniae Alba,Radix Puerariae, Carthamus tinctorius, and so forth.	Gentongping (GTP) granule; 0.32 g/100 g dissolved in saline solution	Male Sprague–Dawley rat	Compression at C6	GTP treatment alleviates spontaneous pain	[4]

Polyphenol	Animal	SCI	Results	Reference
(-)-Epigallocatechin-3-gallate (EGCG); 30 mg/kg (i.p.)	Female Balb/c mice	Contusion at T8-T9	Treatment alleviates thermal hyperalgesia	[5]
(-)-Epigallocatechin-3-gallate (EGCG); 20 mg/kg/h (i.v. during 36 h)	Female Sprague-Dawley rat	Contusion at T9-T10	Treatment alleviates thermal hyperalgesia and mechanical allodynia	[6]

1. Lee, J.Y, Choi, H.Y., Park C,S., Kim, D.H. & Yune, T.Y. Total saponin extract, ginsenoside Rb1, and compound K alleviate peripheral and central neuropathic pain through estrogen receptors on rats. *Phytother Res*. **35**, 2119-2132 (2021).

2. Karami, M. *et al*. S. Crocin improved locomotor function and mechanical behavior in the rat model of contused spinal cord injury through decreasing calcitonin gene related peptide (CGRP). *Phytomedicine*. **21**, 62-7 (2013).

3. Ungerer, G. *et al*. Harpagophytum procumbens Extract Ameliorates Allodynia and Modulates Oxidative and Antioxidant Stress Pathways in a Rat Model of Spinal Cord Injury. *Neuromolecular Med*. **22** ,278-292 (2020).

4. Sun, W. *et al*. Neuroprotective Potential of Gentongping in Rat Model of Cervical Spondylotic Radiculopathy Targeting PPAR-γ Pathway. *J. Immunol. Res*. 2017:9152960 (2017).

5. Álvarez-Pérez, B. *et al*. Epigallocatechin-3-gallate treatment reduces thermal hyperalgesia after spinal cord injury by down-regulating RhoA expression in mice. *Eur. J. Pain*. **20**, 341-52 (2016).

6. Renno, W.M. *et al*. (-)-Epigallocatechin-3-gallate (EGCG) modulates neurological function when intravenously infused in acute and, chronically injured spinal cord of adult rats. *Neuropharmacology*. **77**, 100-19 (2014).
